# Deterministic and Stochastic Allele Specific Gene Expression in Single Mouse Blastomeres

**DOI:** 10.1371/journal.pone.0021208

**Published:** 2011-06-23

**Authors:** Fuchou Tang, Catalin Barbacioru, Ellen Nordman, Siqin Bao, Caroline Lee, Xiaohui Wang, Brian B. Tuch, Edith Heard, Kaiqin Lao, M. Azim Surani

**Affiliations:** 1 Wellcome Trust/Cancer Research UK Gurdon Institute of Cancer and Developmental Biology, University of Cambridge, Cambridge, United Kingdom; 2 Genetic Systems, Applied Biosystems, Life Technologies, Foster City, California, United States of America; 3 Biodynamic Optical Imaging Center, School of Life Sciences, Peking University, Beijing, China; 4 CNRS UMR3215, INSERM U934, Institut Curie, Paris, France; Centro Cardiologico Monzino, Italy

## Abstract

Stochastic and deterministic allele specific gene expression (ASE) might influence single cell phenotype, but the extent and nature of the phenomenon at the onset of early mouse development is unknown. Here we performed single cell RNA-Seq analysis of single blastomeres of mouse embryos, which revealed significant changes in the transcriptome. Importantly, over half of the transcripts with detectable genetic polymorphisms exhibit ASE**,** most notably**,** individual blastomeres from the same two-cell embryo show similar pattern of ASE. However, about 6% of them exhibit stochastic expression, indicated by altered expression ratio between the two alleles. Thus, we demonstrate that ASE is both deterministic and stochastic in early blastomeres. Furthermore, we also found that 1,718 genes express two isoforms with different lengths of 3′UTRs, with the shorter one on average 5–6 times more abundant in early blastomeres compared to the transcripts in epiblast cells, suggesting that microRNA mediated regulation of gene expression acquires increasing importance as development progresses.

## Introduction

Preimplantation development from the totipotent zygote to the blastocyst culminates in the establishment of pluripotent inner cell mass cells (ICM) and trophectoderm cells at embryonic day (E) 3.5 [1]. Differential gene expression in individual cells is a key determinant of cellular differentiation, functions and physiology [2], [3]. This includes allele specific expression (ASE) or differential allelic expression (DAE), which in some instances is due to parent of origin specific imprinting that also affects X inactivation. ASE might also occur due to sequence polymorphisms [4], [5], with consequences for phenotypic diversity and disease susceptibility amongst individuals [6], [7]. A subtle change in gene expression, as in the case of tumor suppressor gene, *Pten,* is sufficient for a profound effect on cell susceptibility phenotype in mice [8]. However, ASE has not been investigated during early development due to the limited amount of material available for analysis. However, it is now feasible to address this question using single cell RNA-Seq analysis.

ASE can potentially serve as an elegant strategy for contributing to cellular diversity with broad functional consequences during development and in adults [9]–[11]. Nearly half of protein-coding genes in humans have multiple alleles with sequence polymorphisms within their mRNA's coding region (CDS) or untranslated region (UTR) [12]. Between 5–50% of the expressed genes exhibit ASE in mammalian tissues or cell lines [13]–[21]. However, it is unclear if different subpopulations of cells at different cell cycle stages or circadian clock stages within a tissue, exhibit the same pattern with respect to ASE (Supporting Information S1). The extent of ASE within individual cells is yet unknown, which is crucial in order to establish how sequence polymorphisms and epigenetic status regulate gene expression differently within the same cell.

ASE when coupled with stochastic gene expression could influence cell phenotype [19]. Seemingly identical cells can be affected by the microenvironment and the intrinsic transcriptional noise, to produce stochastic characteristics [22]–[28], as reflected in the number of mRNA copies from expressed genes [26]. However, the relationship and the extent to which stochasticity contributes to ASE are unknown. Furthermore, this aspect has not been assessed in single blastomeres at the onset of development.

Deep-sequencing based RNA-Seq is a powerful tool to analyze transcriptome at an unprecedented depth and accuracy. We have used single cell RNA-Seq to analyze the digital transcriptome of individual blastomeres in early mouse embryos [29]. We produced over 1 billion 50 base reads from 52 individual mouse cells from oocytes to the blastocyst stage.

## Results

### Developmental path of preimplantation development

On average, our single cell RNA-Seq method detected 14,920 to 18,125 RefSeq transcripts expressed at each stage during preimplantation development (Reads per million, RPM >0.1). On average there were 16,666 transcripts (Table S1) in individual cells, which is more than half of all known genes that are expressed in early development.

**Figure 1:**
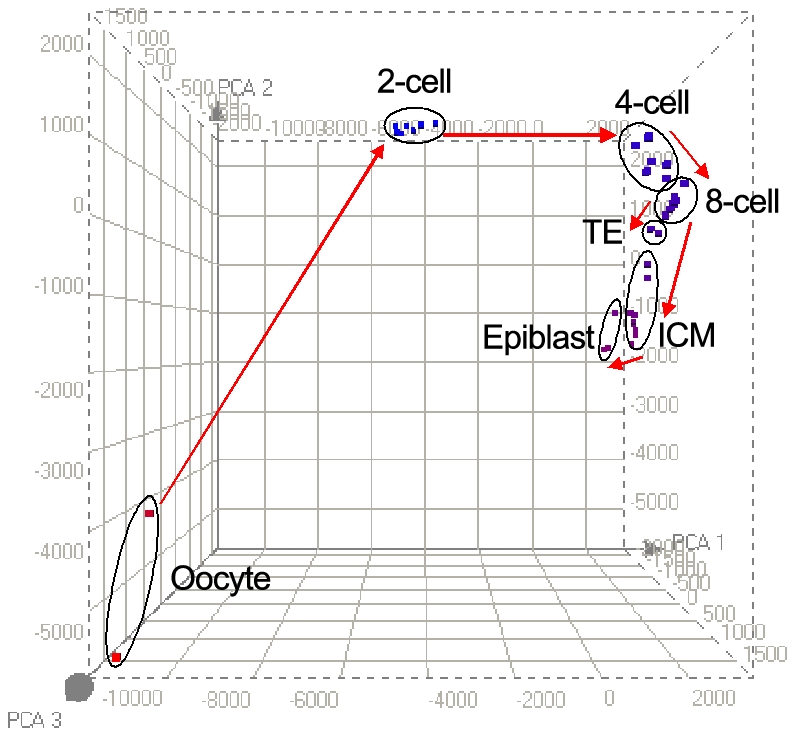
Principal component analysis (PCA) of single cells of preimplantation embryos. Two mature oocytes, eight two-cell blastomeres, six four-cell blastomeres, six eight-cell blastomeres, two trophectoderm cells, nine ICM cells, three epiblast cells are independently clustered.

Using principal component analysis (PCA), we found individual cells to be clustered at each development stage, and the relative distances between each stage represent the extent of change in the transcriptome (Fig. 1). This showed that mature oocytes are most separated from two-cell blastomeres, when the maternal transcriptome is turned off and the zygotic transcriptome is turned on [30]–[33]. We found that 2,193 transcripts were upregulated and 8,173 transcripts downregulated when comparing oocyte with blastomeres from two-cell stage embryos, a change by approximately 70% of all expressed genes. Between two- to four-cell stage, 5,384 transcripts were upregulated, and from four- to eight-cell stage, another 3,412 transcripts were upregulated, which represents the second wave of major zygotic gene activation. Notably, the ‘distance’ in changes from eight-cell blastomeres to the trophectoderm cell (TE) is much shorter than that between eight-cell blastomeres and ICM cells. There is therefore a greater change in the transcriptome from the 8-cell stage to the ICM compared to the change involved in the formation of the TE lineage. This suggests that early blastomeres may be destined for the trophectoderm lineage before diversification towards the pluripotent ICM lineage.

### Comparisons of transcriptomes of individual blastomeres from single preimplantation embryos

There is considerable interest concerning potential differences between individual blastomeres up to the 8-cell stage. We therefore compared different blastomeres within the same embryo [Fold Change, FC(B/B) >2 or <0.5] and between embryos at the same developmental stage [(FC(E/E) >2 or <0.5]. We found that two blastomeres from individual two-cell embryo showed significant similarities [(Pearson correlation coefficient >0.99 (Supporting Information S1)]. By contrast, blastomeres from different embryos at the same developmental stages showed greater differences. This is probably due to cell cycle variations between embryos.

### Allele specific gene expression (ASE) during preimplantation development

**Figure 2:**
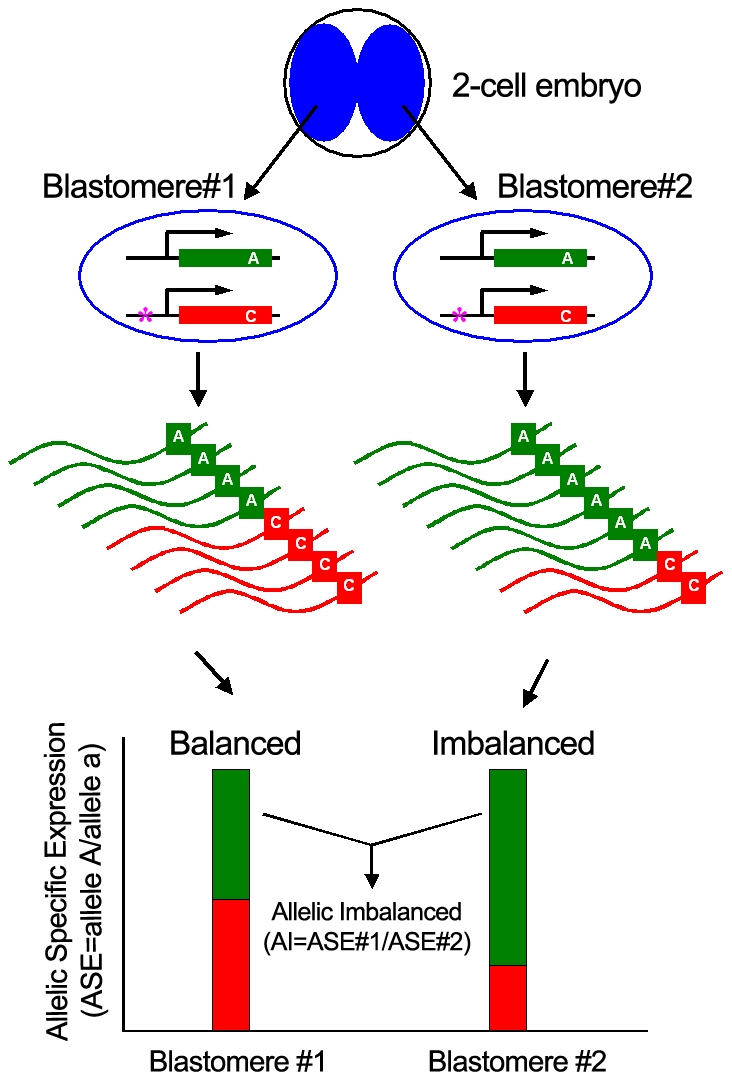
Schematic diagram of allele specific gene expression (ASE) in individual blastomeres of a two-cell embryo identified by SNPs in mRNAs. The two alleles are either expressed equally (balanced as in blastomere #1), or differentially (as in blastomere #2). Allelic ratio = counts of allele A/counts of allele a. If the allelic ratio of the gene is different in the two nearly identical blastomeres from the same two-cell embryo, it is denoted as AI. AI =  (allelic ratio in blastomere #1)/(allelic ratio in blastomere #2). The RNA-Seq reads across the SNP (here using nucleotide ‘A’ and ‘C’ as examples) can discriminate between the two alleles, and reveal relative abundance of expression. Sequence polymorphism is indicated (*).

**Figure 3:**
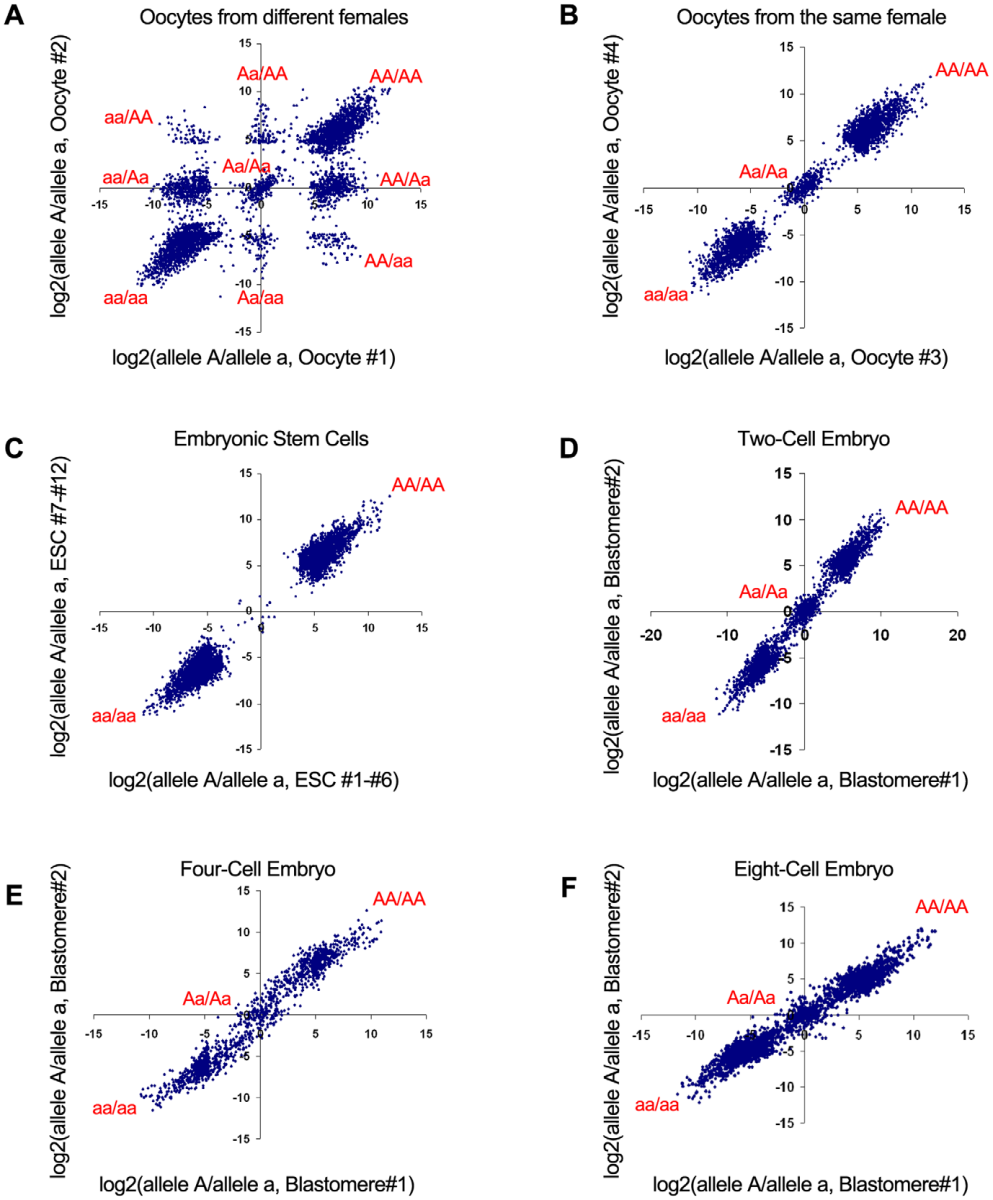
The allelic ratios based on single cell RNA-Seq. Allelic ratios of two mature oocytes (A) obtained from two adult female mice with different genetic background show all 9 possible clusters corresponding to the 9 genotypic combinations between the two cells. Allelic ratios of two mature oocytes obtained from the same adult female mouse (B) show 3 possible genotypes between the two cells: homozygous reference (AA/AA) and alternative (aa/aa) allele, and heterozygous (Aa/Aa). Here ‘A’ means reference allele, whereas ‘a’ means alternative allele. Allelic ratios of twelve nearly homozygous ES cells (C) show the homozygous expression of essentially all (>99.7%) expressed genes. Allelic ratios of two blastomeres of a two-cell stage embryo (D), a four-cell stage embryo (E), and an eight-cell stage embryo (F) show both homozygous and heterozygous loci. To avoid singularity for homozygous loci, we added 1 to the counts when we calculated the allelic ratios.

**Figure 4:**
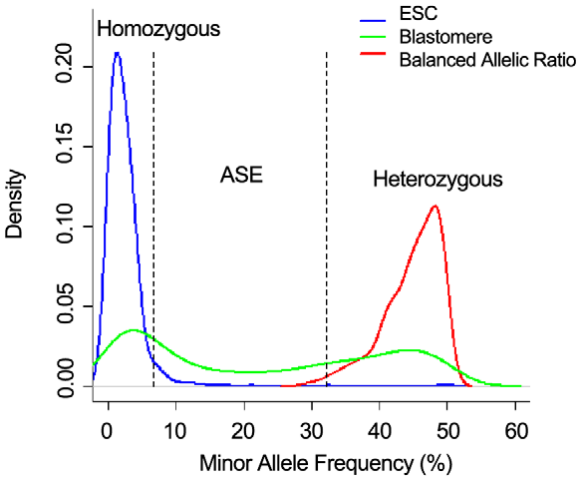
Determination of ASE and AI. Homozygous distribution of the minor allele frequency of 12 mouse ES cells obtained from the same ES cell line, generated from nearly homozygous embryo (blue curve). The distribution of the allele ratio shows that there is a 95% chance for a homozygous locus with at least 25 counts to generate a minor allele frequency of 0.066 or lower. Distribution of minor allele frequency for one blastomere in a two-cell embryo is shown in green. The balanced allele distribution of minor allele frequency for one blastomere from a two-cell embryo is shown in red. The balanced heterozygous expression (equal expression of both alleles) is expected to have their allele frequency of 50%. The distribution generated from all heterozygous loci of an individual cell represents an approximation of the distribution of allelic ratios of balanced loci, that takes in consideration library and instrument biases. Locus is called as having allele specific expression (ASE) if the resulting p-value is <0.01, which corresponds to minor allele frequency of up to 33%, and major allele frequency of at least 67%. The distribution of two-cell blastomere with minor allele frequency above 50% is due to smoothing function produced by R scripts.

We generated over 700 million 50 base reads for 20 individual two- to eight- cell blastomeres to examine the extent and nature of ASE. Our results show that the correlation coefficient between the transcriptomes of two blastomeres from the same two-cell embryo is as high as 0.993 to 0.996, based on the analysis of 16,525 expressed transcripts, which excludes technical bias in our data (Supporting Information S1 and Table S1). To determine ASE in individual blastomeres (Fig. 2 and Supporting Information S1), we examined loci that have two dbSNP (build 128) alleles expressed with a minimum coverage of 25 reads (Fig. 3 and Table S2). To obtain accurate ASE gene calls, we first eliminate homozygous loci that appear to be heterozygous due to potential technical errors by comparing loci from a relatively homozygous mouse embryonic stem (ES) cell line, where 95% of the time the minor allele frequency accounts for less than 6% (Fig. 4, Supporting Information S1). After filtering our data for homozygous loci, we found that between 531 and 2,029 of them in individual cells have expressed heterozygous SNPs (Tables 1 and 2 and Table S2), which allowed us to discriminate between the mRNA derived from both alleles of a gene in a single cell.

**Table 1:** Allelic specific expression (ASE), allelic imbalance (AI) determination, and false positive rate for oocytes and blastomeres in 2-cell embryos.

ASE and AI determination	Oocyte_1	Oocyte_2	Oocyte_3	Oocyte_4	2cell_E1_B1	2cell_E1_B2	2cell_E2_B1	2cell_E2_B2	2cell_E3_B1	2cell_E3_B2	2cell_E4_B1	2cell_E4_B2
selected loci*	2312	2537	1358	1357	1640	1645	1308	1586	974	1827	1253	1546
# of Heterozygous	2029	1853	911	905	1042	984	761	931	531	1021	677	856
# of ASE	483	589	198	146	418	433	299	405	228	412	304	444
# False Positive (ASE)	35	55	33	33	42	45	36	44	29	53	37	45
# Allelic Imbalance (AI)	1402	1402	39	39	60	60	53	53	32	32	49	49
# False Positive (AI)	7.28	9.26	16.48	22.49	10.02	10.31	12.17	10.81	12.56	12.77	12.20	10.11
ASE (%)	23.80	31.79	21.73	16.13	40.12	44.00	39.29	43.50	42.94	40.35	44.90	51.87
AI (%)	72.23	72.23	4.30	4.30	5.92	5.92	6.26	6.26	4.12	4.12	6.39	6.39

**Table 2:** Allelic specific expression (ASE), allelic imbalance (AI) determination, and false positive rate for blastomeres in 4-cell and 8-cell embryos.

ASE and AI determination	4cell_E1_B1	4cell_E1_B2	4cell_E2_B1	4cell_E2_B2	4cell_E3_B1	4cell_E3_B2	8cell_E1_B1	8cell_E1_B2	8cell_E2_B1	8cell_E2_B2	8cell_E3_B1	8cell_E3_B2
selected loci*	1806	2793	700	2821	896	3077	2715	1787	2482	2409	2514	2315
# of Heterozygous	1186	1583	448	1802	574	1935	1493	989	1206	1257	1429	1419
# of ASE	821	1072	286	1156	328	1091	940	645	714	802	965	916
# False Positive (ASE)	44	80	18	72	23	79	79	52	79	73	71	61
# Allelic Imbalance (AI)	139	139	75	75	190	190	118	118	161	161	166	166
# False Positive (AI)	5.42	7.42	6.20	6.20	6.92	7.28	8.43	8.04	11.09	9.13	7.40	6.70
ASE (%)	69.22	67.72	63.84	64.15	57.14	56.38	62.96	65.22	59.20	63.80	67.53	64.55
AI (%)	10.04	10.04	6.67	6.67	15.15	15.15	9.51	9.51	13.07	13.07	11.66	11.66

The locus counts of ASE and AI in individual cells of early embryos. Loci were selected based on the following criteria: (1) Sequence data produced multiple nucleotides; (2) Observed alleles are listed in dbSNP; (3) The position is covered by at least 25 uniquely aligned reads; (4) No more than 50% of covering reads supporting each nucleotide align to identical positions in the genome; (5) UCSC (University of California Santa Cruz) annotation supports transcription of only one strand.

Between 39 and 51% of the selected heterozygous loci show clear ASE in individual cells of a two-cell embryo (Tables 1 and 2, Fig. 4, Supporting Information S1). Thus, the two alleles of a significant number of genes in the same individual cell exposed to identical trans-regulating factors respond differentially and are expressed at different levels. This differential response to an identical intracellular environment potentially provides relatively stable, yet flexible expression by allowing the two alleles to respond differently to trans-acting signals/factors within the same cell [9]. An alternative possibility is that allelic differences in *cis* regulatory elements might have an important impact on the activity of many genes that we decided to explore further.

Differential allelic expression might be predetermined by genetic differences such as *cis* regulatory elements or epigenetic modifications such as local chromatin structure, or these differences may arise randomly affecting either allele. If ASE is predetermined, we would expect to find an essentially identical pattern in individual blastomeres of a two-cell embryo. Using SNP-coupled genetic polymorphism to identify the alleles of individual genes, we found that for more than ninety percent of the genes in two-, and four-cell embryos, expression of one of the alleles, henceforth called allele *A*, was always higher than that of the other allele *a* in individual cells within the same embryo (Table S2). Thus, for nearly half of the expressed genes distinguishable by mRNA SNPs, their functional read out was coupled to genetic polymorphism in *cis* in early embryos. This indicates that trans-regulatory factors such as differences in cell cycle, asymmetric division or micro-environment, do not have a significant effect. The differential allelic expression might be regulated either directly through deployment of the transcription machinery, or indirectly by differential epigenetic marking and status of the two alleles in the same cell [34]–[36]. The ability of two alleles to respond differently within the same cell offers a possible explanation for why progeny from out bred crosses with two distinct alleles often show superior performance compared with those from inbred parents, since the choice of alternative alleles might be advantageous through robustness of response to the environment [34].

Next we addressed how genetic background affects regulation of gene expression, since we had obtained embryos from MF1 out bred mice for this analysis. We found that for gene loci sharing the same set of heterozygous alleles across blastomeres at the same developmental stage, but from different embryos and therefore with different genetic backgrounds, between 66% and 83% showed allelic ratios that were skewed in the same direction (Table S2). This implies that a large number of alleles are systematically expressed more highly within individual cells of different embryos. Nonetheless, since some of the loci can show ASE in either direction, this indicates that genetic background can also sometimes affect the allele that is predisposed for expression within individual cells.

Next we asked if stochastic events contribute to differential allelic expression. For this, it is necessary to distinguish between ‘noise’ that could be due to technical issues, from genuine stochastic differences in expression, particularly as both might occur on a similar scale. To eliminate differences in expression due to technical reasons, we looked at the expression ratios of two alleles of genes in individual blastomeres obtained from the same two-cell embryos, which will potentially remove all technical noise associated with similar cells from different embryos (Fig. 2). We found that for nearly 6% of the expressed loci with distinguishable heterozygous alleles at the two-cell stage, and 10–12% of them at the four-cell and eight-cell stage, there was a significant difference in the magnitude of allele specific expression in blastomeres from the same embryo (allelic imbalance (AI), FC[(allele A/allele a in blastomere #1)/(allele A/allele a in blastomere #2)] >2 or <0.5, p<0.05) (Table 1). Thus, there could be hundreds of genes in early embryos with intrinsic stochastic characteristics, which as expected have no concordance in similarly analyzed blastomeres from different two-cell embryos (Table S3). Thus, combined data leads to a critical conclusion that for hundreds of genes, the combination of deterministic and stochastic expression establishes their functional readout, which can potentially control cell fate, developmental potential and phenotype of early embryo and adult organisms. Furthermore, these observations might offer an explanation, at least in part, for the developmental differences between identical twins, beyond environmental effects.

### Alternative splicing during preimplantation development

Alternative splicing is known to play an important role in defining cell identity and their physiological function [37]. We detected 18–25% of known transcript variants (RPM >0.1) (Table S4). From oocyte to two-cell stage, 269 transcript variants were up regulated (fold change, FC[oocyte/two-cell, splicing]>2, p<0.05), and 677 variants were down regulated (FC[oocyte/two-cell, splicing]<0.5, p<0.05). There are even more transcript variants from two- to four-cell stage, but less so from eight-cell to blastocyst stage. Next we asked if different transcript isoforms of a gene show similar changes between different developmental stages, or if these changes are significantly different. We detected dramatic differential regulation of simultaneously expressed transcript variants during preimplantation development. (Table S4). Some of these transcript variants can encode different proteins by skipping exons or by utilizing alternative exons, suggesting that some proteins from a gene may have different expression dynamics during early embryonic development.

### Dynamic changes of 3′UTR lengths during preimplantation development

**Figure 5:**
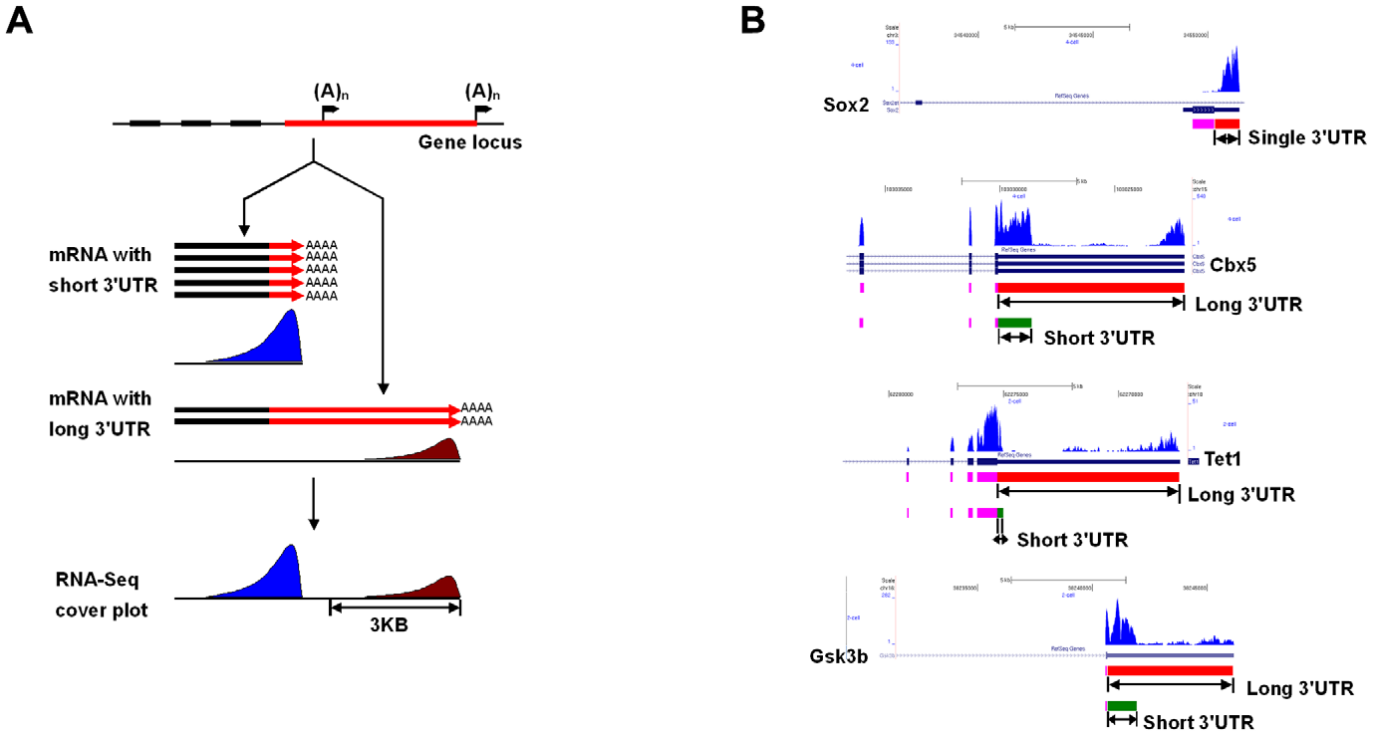
Schematic diagrams (A) and coverage plots (B) that express short and long 3′UTR isoforms. Co-expression of two types of mRNAs of long (distal) and short (proximal) 3′UTR from the same gene, such as Cbx5, Tet1, and Gsk3b. Here use Sox2 as a reference, which has only one short 3′UTR. The 3′ bias of single cell RNA-Seq method can discriminate between the long and short 3′UTRs of the mRNAs from individual genes by looking at the shape and the spread of the cover plot of an expressed gene.

Our analysis also revealed differences in the 3′UTRs of transcripts [38]–[41]. Whereas these differences do not appear to have any relationship with ASE, they might contribute to the regulation of gene expression. The single cell RNA-Seq analysis uses poly(T) primer for reverse transcription, which is biased towards a better coverage of the 3′ end of mRNAs, so that after about 1 kb from 3′end of mRNAs, the read coverage dropped significantly and nearly disappeared after 1.5 kb (Fig. 5 and Table S5). However, for a large number of genes with long 3′UTR at the region upstream of the 1.5 kb of 3′ most regions, there are other peaks of reads covering the 5′ part of 3′UTRs and often with significantly more coverage than the distal 3′UTR (Fig. 5B and Supporting Information S1). We propose that this is due to the presence of alternative transcripts from the same gene with shorter 3′UTR. We found that 1,718 genes have two transcript isoforms with the same coding region but different lengths of 3′UTR (Table S5), suggesting that approximately 13% of expressed genes exhibit at least two isoforms with long (distal) and short (proximal) 3′UTR in the same cell at the same time (Supporting Information S1). We verified the RNA-Seq result with allele specific RT-qPCR (Supporting Information S1, Table S6, and Table S7).

**Figure 6:**
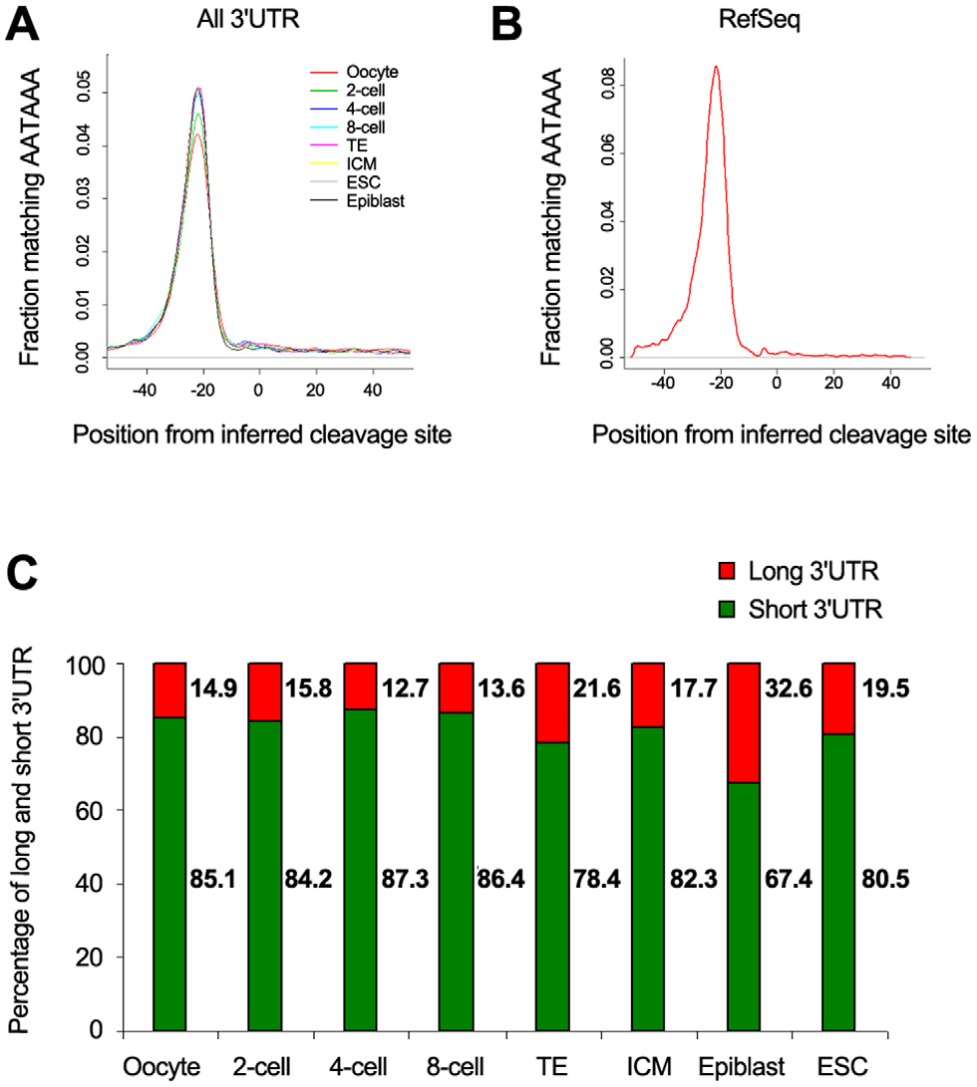
Features of alternative 3′UTRs during preimplantation development. Density plots of CPSF binding motif distance from predicted 3′UTR site. For all cell stages analyzed, CPSF binding motifs are observed 20 nt upstream of the predicted cleavage site (A) as expected, in concordance with CPSF location relative to known RefSeq cleavage sites (B). (C) The ratio of expression levels between the proximal (short) and distal (long) 3′UTR of the same genes during preimplantation development.

**Figure 7:**
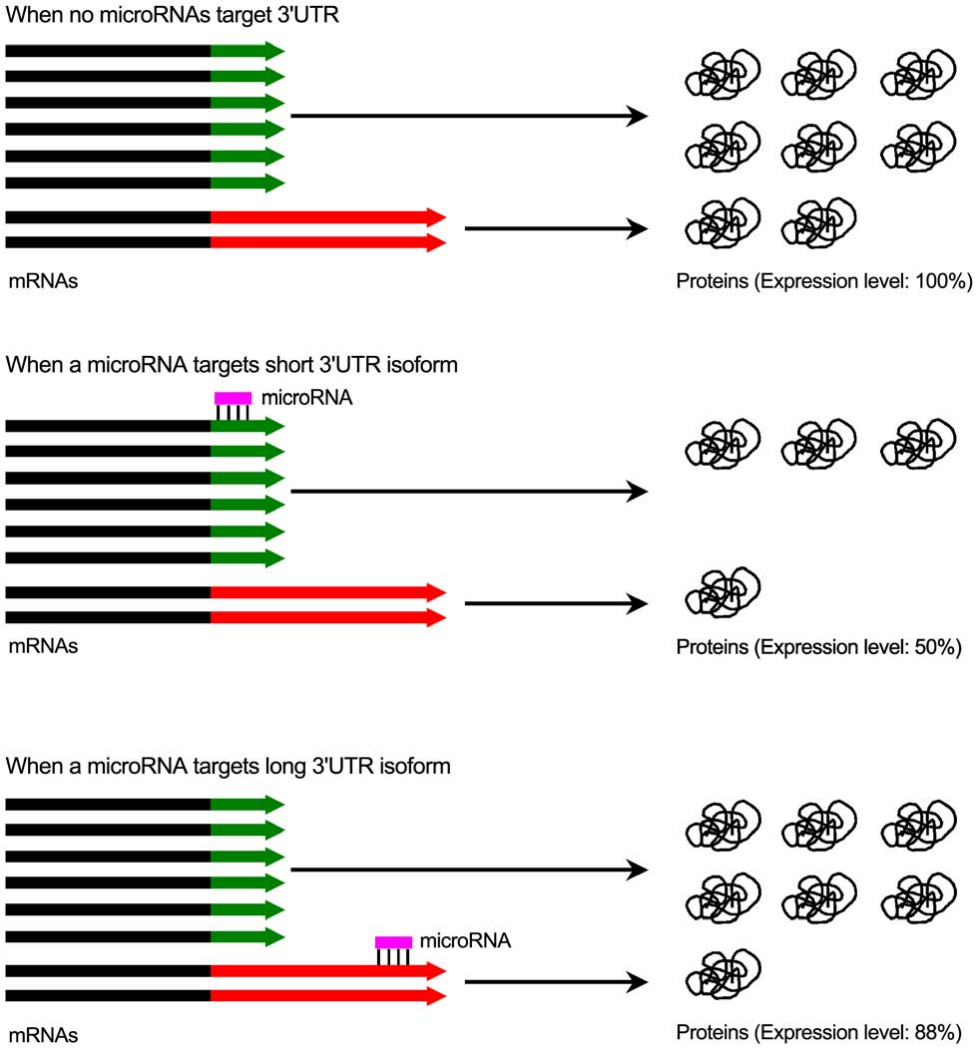
Potential regulation of long and short 3′UTRs of the same gene within individual cells by microRNAs.

The majority of transcripts with different 3′UTRs from a gene are due to alternative polyadenylation, since 40% of short (proximal) and 70% of long (distal) 3′UTRs, respectively, have CPSF binding sites located at −20 nt upstream of their 3′end, suggesting that the stop of the majority of these 3′UTRs are at the canonical polyadenylation recognition sites (Fig. 6). We propose that in individual blastomeres, the majority of transcripts from a gene have a short 3′UTR, which will be translated constantly, whereas for a small number of transcripts from the same gene with long 3′UTR, their translation will be dynamically regulated by microRNAs or RNA-binding proteins (Fig. 7). This would allow relatively stable translation of a large set of genes, while permitting subtle adjustments in protein levels. The abundance of transcript isoforms with short 3′UTR is about 5–6 fold higher compared with those from the same gene with a long 3′UTR in two-cell embryos (Fig. 6C and Supporting Information S1). Thus, the impact of miRNA regulation of mRNA translation for these mRNAs with two different lengths of 3′UTRs in early blastomeres is probably negligible (Fig. 7).

Interestingly, whereas the ratio of long to short 3′UTR is maintained at four- and eight-cell stages, it changes in postimplantation epiblast cells, where mRNAs with the longer 3′UTR isoforms increase from 16% to 33% (Fig. 6). This suggests that later in development, transcripts with long 3‘UTR might provide greater flexibility for translational regulation, at a time when cells respond to more complex signaling conditions for diverse cell fate decisions.

### Expression dynamics of long non-coding RNAs

Non-coding RNAs play an important role during early embryonic development [42]. We found that for the 1,446 non-coding genes in the RefSeq database, between 400 and 600 of them were expressed during preimplantation development of which, on average, 203 of them showed dynamic changes, suggesting that they may contribute to the regulation of early development (Table S1). Furthermore, 3′UTR of non-coding RNAs increased significantly from mature oocyte to two-cell blastomeres, but decreased slightly from two- to four- to eight-cell stage (Table S5). This was maintained thereafter in the ICM but decreased significantly in TE. At E4.5, the length of 3′UTR decreased significantly in the epiblast, which matches with the pattern for the protein-coding genes. This shows that as with the protein-coding genes, non-coding RNAs have similar pattern of expression level dynamics and of the length of 3′UTR.

## Discussion

A crucial step towards understanding early embryonic development is to obtain comprehensive knowledge of gene expression network underlying the ordered development phenotype. Previously the transcriptome landscape of preimplantation embryos has been analyzed using hundreds of pooled embryos. Furthermore, there is as yet no complete description of the overall differences in the transcriptomes of the ICM and trophectoderm. We have taken advantage of our single cell RNA-Seq analysis of blastomeres to address some of these questions (Table S8). We found that the most dramatic change in gene expression network occurred between oocyte and two-cell embryo stage when the maternal transcriptome is replaced by the expression of the zygotic transcriptome. We also found that eight-cell blastomeres are clearly more similar to trophectoderm cells than to ICM cells, suggesting that the initial development of blastomeres is probably towards the trophectoderm lineage from which the pluripotent ICM lineage emerges subsequently. We also obtained comprehensive transcriptomes of the ICM and trophectoderm.

It may be argued that changes in expressed genes may not necessarily reflect corresponding protein abundance due to extensive post-transcriptional and post-translational regulation. However, global transcriptome and proteome analysis has shown that transcriptomes exhibit strong correlation with corresponding proteomes [43]. In addition, we also detected significant numbers of long non-coding RNAs. Nearly half of these non-coding RNAs showed dynamic changes during preimplantation development, which not only reflects the overall transcriptional activity of the genome, but it also reflects the functional status of the genome during early development.

Concerning ASE, over half of the genes with distinguishable SNPs display differential ASE in individual cells. Notably, a significant number of these specific alleles are systematically more highly expressed in blastomeres from different embryos, indicating that differences in their *cis*-regulatory elements may play an important role in determining allele-specific expression. Future studies with hybrid embryos from mutual crossing of two different strains of inbred mice will be required to obtain further insights into the influence of the parental origin of genes and *cis-­*regulatory elements in dictating ASE. However, we also found several genes, which show intrinsic stochastic expression characteristics that may contribute to the differences between identical cells. Such differences could contribute to phenotypic differences, and account for the divergence between identical twins, independently of genetic and environment factors. We cannot formally exclude the possibility that the high percentage of ASE within individual early blastomeres is due to the presence of maternally inherited transcripts. However this seems unlikely, because if this were the case, the subsequent decline in the maternal transcripts during early development should result in a significant reduction in ASE. However, we see an increase in the percentage of ASE between 2 and 8-cell stage. We cannot rule out transcriptional oscillation as a contributing factor to ASE, particularly for those genes where the ASE is shifted to one allele or the other between blastomeres.

We also found that the lengths of the 3′UTR of transcripts show surprising changes during development, indicating that for many genes there are also post-transcriptional control mechanisms, which impact on translational regulation during early development. A large number of transcripts are known to be the targets of microRNAs [44]. Here we found that nearly two thousand genes with potential microRNA binding sites have majority of their mRNA copies with truncated 3′UTRs, which would potentially allow them to escape from possible repression by the co-expressed microRNAs (Fig. 7). This is most significant up to the eight-cell stages. The proportion of transcripts with longer 3′UTRs increased later, which probably allows for the establishment of more complex gene regulation networks as development progresses, through more flexible regulation of mRNAs by microRNAs, and possibly by RNA binding proteins.

## Materials and Methods

### Isolation of Mouse Early Embryos and Single Blastomeres

All the mouse work has been conducted under the license from Home Office of UK (the project license number is: PPL 80/2193). All embryos at two-cell, four-cell, and eight-cell stages were recovered from outbred MF1 females mated with MF1 male mice [45]. Two-cell, four-cell, and eight-cell embryos were flushed from the oviduct using a 30-gauge blunt tip flushing needle at E1.5, E2.0, and E2.5, respectively. The zona pellucida was removed by use of acidic tyrode solution. Then the individual blastomeres were separated by gentle pipetting with a glass capillary in calcium-free medium. The resulting single cells were washed twice in 0.1% BSA in 1X PBS before they were picked individually for subsequent analysis.

### Preparation of single cell cDNAs

The single-cell RNA-seq was performed following a detailed protocol we developed recently [46], [47]. Briefly, an individual cell was manually picked and transferred into lysate buffer by a glass capillary controlled by a mouth pipette. The reverse transcription was directly done using the whole cell lysate. Then a poly(A) tail was added to the 3′ end of first-strand cDNAs using terminal deoxynucleotidyl transferase, which was followed by 20 plus 9 cycles of PCR to amplify the single cell cDNAs evenly.

### Alignment and algorithm

Libraries produced from individual cells were barcoded and sequenced using the SOLiD system. The 60 Gb of sequence produced by 50 bp reads was aligned (www.solidsoftwaretools/gf/project/transcriptome/, raw data was deposited in GEO database, GSE22182) to the mouse reference genome (NCBI build 37) containing IUB codes on every locus reported in dbSNP database (build 128), in order to minimize the coverage bias of the reference allele. For each alignment location we assign a score that reflects the size of alignment and the number of mismatches between reads and the reference genome. For each read, low scoring alignments (more than 9 units lower than the maximum alignment score of the read) are removed and uniquely aligned reads are used for downstream analysis (Table S1, Supporting Information S1). For more details of alignment scoring, see [29].

### Allelic expression

For a given genomic coordinate, the “allelic ratio” is the number of reads aligned across a given position which contains the reference nucleotide divided by the number of reads containing the first non-reference nucleotide in dbSNP (Supporting Information S1). Thus we will only concern ourselves here with the subset of genomic positions for which (1) sequence data produces multiple nucleotides; (2) observed alleles are listed in dbSNP; (3) the position is covered by at least 25 uniquely aligned reads; (4) no more than 50% of the covering reads supporting each nucleotide align to identical positions in the genome (in order to account for PCR replication); and (5) UCSC annotation supports transcription of only one strand (Table S2 and Supporting Information S1). These genomic positions account for less than 1% of all sequenced bases, while the remaining 99% of the sequenced bases show a single nucleotide, indicating that those loci are homozygous (and so are not included for allele specific expression).

### Allele specific expression (ASE)

In general, a complete solution for this problem is difficult to answer with only RNA libraries [14]. There are three essential advantages to this study that allows us to produce such a solution: (1) our libraries are produced from single cells, which makes every SNP either homozygous or heterozygous; (2) the use of cells produced from nearly inbred mice as a negative control (Table S2 and Supporting Information S1), which enables the detection of heterozygous loci; and (3) double-strand cDNA synthesis, which enables us to measure the variation of allelic ratios from balanced loci. The method we used here is based on empirical distributions calculated from data and therefore incorporates both library and instrument biases.

For every locus of interest (as described in the beginning of this section), we want to use allelic ratios for the common alleles based on the NCBI dbSNP annotation to detect heterozygous loci and determine the allelic specific expression of the 2 alleles. Detection of heterozygous loci is mainly affected by errors produced in reverse transcription combined with PCR amplification steps, which are known to create false polymorphic sites. Additionally, instrument error, even after 2 base encoding corrections, can produce the same effect.

In order to measure the extent of these biases, we used 12 mouse ES cells (for one of these cells, we have two technical replicates) obtained from the same embryonic stem cell line, generated from a nearly inbred (near homozygous) embryo. All of the 12 ES cells have identical genomic background, which allows us to aggregate data from all cells, and additionally, they are known to be nearly homozygous (Table S3). The allelic ratios generated from these cells help us measure the biases mentioned above. Figure S5 shows that there is a 95% chance for a homozygous locus in the search space to generate a minor (false) allele frequency of 0.066 or less. We use this observation to call a locus (from search space) heterozygous if the minor allele frequency is above 0.066. The expected false positive rate of incorrectly calling a homozygous locus as a heterozygous one will be less than 5% (Supporting Information S1). We also designed allele specific real-time PCR assays and validated 23 out of 26 ASE loci for 20 individual blastomeres (Supporting Information S1, Table S7)

Next, we want to measure the variation in allelic ratios of heterozygous loci introduced by library preparation and instrument measurement. To do this, consider that we have a SNP with 2 alleles (at the cDNA level): a_1_ = 5′ ATC**G**CCC 3′ and a_2_ = 5′ ATC**A**CCC 3′ that have x and y copies, respectively. In the library preparation, the second strand is synthesized and x and y copies of the alleles b_1_ = 3′ TAG**C**GGG 5′ and b_2_ = 3′ TAG**T**GGG 5′ are complementarily produced. The ratio B =  (number of a_1_ observations + number of b_2_ observations)/(number of a_2_ observations + number of b_1_ observations) should equal 1 if no library and instrument biases exists, irrespective if the locus is allelic balanced or not (Supporting Information S1). Since the alignment method we used makes a distinction between the two strands of the mouse genome, the number of a_1_ allele observations is equal to the number of reads aligned to the positive strand containing nucleotide G (same for all other alleles). The distribution of this statistic D generated from all heterozygous loci of an individual cell represents an approximation of the distribution of allelic ratios of balanced loci, which takes into consideration library and instrument biases. For a locus with allelic ratio r, the associated p-value (when testing H_0_: locus has balanced allelic expression) is the result of the two-tailed test when r is generated from D distribution. The locus is called as having allele specific expression (ASE) if the resulting p-value <0.01 (Supporting Information S1, and Tables 1 and 2).

### Allelic expression imbalance

Next we investigated the extent and types of allelic imbalance (AI) observed between blastomeres from the same embryos (AI =  (ASE in blastomere #1)/(ASE in blastomere #2)). Here we focus on relative AI, which compares the ratio of the expression of two alleles between pairs of blastomeres from same embryo [48]. We analyzed further only those genomic positions having a significant AI with χ^2^ p-value less than 0.05 (Tables 1 and 2 and Table S3).

## Supporting Information

Table S1 Single cell RNA-Seq counts and RPM mapped to RefSeq of single oocytes, blastomeres of pre-implantation embryos.
(TXT)

Table S2 Allelic Specific Expression (ASE) of single oocytes, and blastomeres of 2-cell, 4-cell, and 8-cell stage embryos. Each entry represents a locus that was found to be heterozygous in at least half of the cells of one cell type. For each such locus we generate observed allele counts, log2 allelic ratio and p-value (as described in SOM) from each individual cell.
(TXT)

Table S3 ASE counts and AI χ^2^ test *p*-values for ES cells, and 2-cell, 4-cell, 8-cell embryos. The log2(AI)  = log2[(ASE in blastomere #1)/(ASE in blastomere #2)] and χ^2^ *p*-values were calculated for 12 ES cells and each blastomeres.
(XLS)

Table S4 Single cell RNA-Seq counts and RPM mapped to unique known exon-exon junctions and exons for each known splicing variant in RefSeq of single oocytes, blastomeres of pre-implantation embryos.
(XLS)

Table S5 Consensus 3′UTRs of single mature oocytes, blastomeres of 2-cell, 4-cell, and 8-cell stage embryos, TE, and ICM of E3.5 blastocysts, epiblast of E4.5 blastocysts, and ESC. Each entry presents up to six UTR predictions of a RefSeq transcript. For each cell type we report a predicted UTR size(s) if the prediction was observed in at least half of all the individual cells.
(TXT)

Table S6 The expression of 380 genes in blastomeres of 2-cell and 4-cell embryos by single cell TaqMan real-time PCR.
(XLS)

Table S7 Allelic specific real-time PCR validation of ASE based on single cell RNA-Seq data. The averaged Ct values and standard deviations (SD) were calculated from three technical repeats.
(XLS)

Table S8 Gene Ontology (GO) analysis results of functions, networks, and components for genes with their fold changes more than 2-fold and p-values <0.01 between two developmental stages.
(XLS)

Supporting Information S1 Supporting materials including supplementary discussions, methods, figures, and references.
(PDF)

## References

[1] Rossant J, Tam PP (2009). Blastocyst lineage formation, early embryonic asymmetries and axis patterning in the mouse.. Development.

[2] Cheung VG, Spielman RS (2002). The genetics of variation in gene expression.. Nat Genet.

[3] Cheung VG, Bruzel A, Burdick JT, Morley M, Devlin JL (2008). Monozygotic twins reveal germline contribution to allelic expression differences.. Am J Hum Genet.

[4] Chow J, Heard E (2009). X inactivation and the complexities of silencing a sex chromosome.. Curr Opin Cell Biol.

[5] Ferguson-Smith AC, Surani MA (2001). Imprinting and the epigenetic asymmetry between parental genomes.. Science.

[6] Montgomery SB, Sammeth M, Gutierrez-Arcelus M, Lach RP, Ingle C (2010). Transcriptome genetics using second generation sequencing in a Caucasian population.. Nature.

[7] Pickrell JK, Marioni JC, Pai AA, Degner JF, Engelhardt BE (2010). Understanding mechanisms underlying human gene expression variation with RNA sequencing.. Nature.

[8] Alimonti A, Carracedo A, Clohessy JG, Trotman LC, Nardella C (2010). Subtle variations in Pten dose determine cancer susceptibility.. Nat Genet.

[9] Gregg C, Zhang J, Weissbourd B, Luo S, Schroth GP (2010). High-resolution analysis of parent-of-origin allelic expression in the mouse brain.. Science.

[10] Gregg C, Zhang J, Butler JE, Haig D, Dulac C (2010). Sex-specific parent-of-origin allelic expression in the mouse brain.. Science.

[11] McDaniell R, Lee BK, Song L, Liu Z, Boyle AP (2010). Heritable individual-specific and allele-specific chromatin signatures in humans.. Science.

[12] Levy S, Sutton G, Ng PC, Feuk L, Halpern AL (2007). The diploid genome sequence of an individual human.. PLoS Biol.

[13] Yan H, Yuan W, Velculescu VE, Vogelstein B, Kinzler KW (2002). Allelic variation in human gene expression.. Science.

[14] Zhang K, Li JB, Gao Y, Egli D, Xie B (2009). Digital RNA allelotyping reveals tissue-specific and allele-specific gene expression in human.. Nat Methods.

[15] Pant PV, Tao H, Beilharz EJ, Ballinger DG, Cox DR (2006). Analysis of allelic differential expression in human white blood cells.. Genome Res.

[16] Bjornsson HT, Albert TJ, Ladd-Acosta CM, Green RD, Rongione MA (2008). SNP-specific array-based allele-specific expression analysis.. Genome Res.

[17] Gagneur J, Sinha H, Perocchi F, Bourgon R, Huber W (2009). Genome-wide allele- and strand-specific expression profiling.. Mol Syst Biol.

[18] Main BJ, Bickel RD, McIntyre LM, Graze RM, Calabrese PP (2009). Allele-specific expression assays using Solexa.. BMC Genomics.

[19] Palacios R, Gazave E, Goñi J, Piedrafita G, Fernando O (2009). Allele-specific gene expression is widespread across the genome and biological processes.. PLoS One.

[20] Heap GA, Yang JH, Downes K, Healy BC, Hunt KA (2010). Genome-wide analysis of allelic expression imbalance in human primary cells by high-throughput transcriptome resequencing.. Hum Mol Genet.

[21] Daelemans C, Ritchie ME, Smits G, Abu-Amero S, Sudbery IM (2010). High-throughput analysis of candidate imprinted genes and allele-specific gene expression in the human term placenta.. BMC Genet.

[22] Arias AM, Hayward P (2006). Filtering transcriptional noise during development: concepts and mechanisms.. Nat. Rev. Genet.

[23] Raj A, van Oudenaarden A (2008). Nature, nurture, or chance: stochastic gene expression and its consequences.. Cell.

[24] Huang S (2009). Non-genetic heterogeneity of cells in development: more than just noise.. Development.

[25] Chang HH, Hemberg M, Barahona M, Ingber DE, Huang S (2008). Transcriptome-wide noise controls lineage choice in mammalian progenitor cells.. Nature.

[26] Raj A, Peskin CS, Tranchina D, Vargas DY, Tyagi S (2006). Stochastic mRNA synthesis in mammalian cells.. PLoS Biol.

[27] Cohen AA, Geva-Zatorsky N, Eden E, Frenkel-Morgenstern M, Issaeva I (2008). Dynamic proteomics of individual cancer cells in response to a drug.. Science.

[28] Taniguchi Y, Choi PJ, Li GW, Chen H, Babu M (2010). Quantifying E. coli proteome and transcriptome with single-molecule sensitivity in single cells.. Science.

[29] Tang F, Barbacioru C, Wang Y, Nordman E, Lee C (2009). mRNA-Seq whole-transcriptome analysis of a single cell.. Nat Methods.

[30] Sharov AA, Piao Y, Matoba R, Dudekula DB, Qian Y (2003). Transcriptome analysis of mouse stem cells and early embryos.. PLoS Biol.

[31] Hamatani T, Carter MG, Sharov AA, Ko MS (2004). Dynamics of global gene expression changes during mouse preimplantation development.. Dev Cell.

[32] Wang QT, Piotrowska K, Ciemerych MA, Milenkovic L, Scott MP (2004). A genome-wide study of gene activity reveals developmental signaling pathways in the preimplantation mouse embryo.. Dev Cell.

[33] Bell CE, Calder MD, Watson AJ (2008). Genomic RNA profiling and the programme controlling preimplantation mammalian development.. Mol Hum Reprod.

[34] O'Neill MJ (2005). The influence of non-coding RNAs on allele-specific gene expression in mammals.. Hum Mol Genet.

[35] Kadota M, Yang HH, Hu N, Wang C, Hu Y (2007). Allele-specific chromatin immunoprecipitation studies show genetic influence on chromatin state in human genome.. PLoS Genet.

[36] Schalkwyk LC, Meaburn EL, Smith R, Dempster EL, Jeffries AR (2010). Allelic skewing of DNA methylation is widespread across the genome.. Am J Hum Genet.

[37] Chen M, Manley, L J (2009). Mechanisms of alternative splicing regulation: insights from molecular and genomics approaches.. Nat Rev Mol Cell Biol.

[38] Sandberg R, Neilson JR, Sarma A, Sharp PA, Burge CB (2008). Proliferating cells express mRNAs with shortened 3′ untranslated regions and fewer microRNA target sites.. Science.

[39] Mayr C, Bartel DP (2009). Widespread shortening of 3′UTRs by alternative cleavage and polyadenylation activates oncogenes in cancer cells.. Cell.

[40] Ji Z, Tian B (2009). Reprogramming of 3′ untranslated regions of mRNAs by alternative polyadenylation in generation of pluripotent stem cells from different cell types.. PLoS One.

[41] Ji Z, Lee JY, Pan Z, Jiang B, Tian B (2009). Progressive lengthening of 3′ untranslated regions of mRNAs by alternative polyadenylation during mouse embryonic development.. Proc Natl Acad Sci U S A.

[42] Guttman M, Amit I, Garber M, French C, Lin MF (2009). Chromatin signature reveals over a thousand highly conserved large non-coding RNAs in mammals.. Nature.

[43] Fu X, Fu N, Guo S, Yan Z, Xu Y (2009). Estimating accuracy of RNA-Seq and microarrays with proteomics.. BMC Genomics.

[44] Stark A, Brennecke J, Bushati N, Russell RB, Cohen SM (2005). Animal MicroRNAs confer robustness to gene expression and have a significant impact on 3′UTR evolution.. Cell.

[45] Nagy A, Gertsenstein M, Vintersten K, Behringer R (2003). Recovery and in vitro culture of preimplantation stage embryos.. In: Manipulating the Mouse Embryo 3rd edn.

[46] Tang F, Barbacioru C, Bao S, Lee C, Nordman E (2010). Tracing the derivation of embryonic stem cells from the inner cell mass by single-cell RNA-Seq analysis.. Cell Stem Cell.

[47] Tang F, Barbacioru C, Nordman E, Li B, Xu N (2010). RNA-Seq analysis to capture the transcriptome landscape of a single cell.. Nat Protoc.

[48] Tuch BB, Laborde RR, Xu X, Gu J, Chung CB (2010). Tumor transcriptome sequencing reveals allelic expression imbalances associated with copy number alterations.. PLoS One.

