# Supplementary material for: Deterministic and Stochastic Allele Specific Gene Expression in Single Mouse Blastomeres
Source: PLoS One. 2011 Jun 23;6(6):e21208. doi: 10.1371/journal.pone.0021208 (PMC3121735; doi:10.1371/journal.pone.0021208)
Supplement: Supporting Information S1 — Supporting materials including supplementary discussions, methods, figures, and references. (PDF) [file pone.0021208.s009.pdf]

SUPPORTING INFORMATION S1

SUPPLEMENTARY DISCUSSIONS

Gene Ontology analysis of preimplantation development

We did Gene Ontology (GO) analysis for the genes showing dynamic expression patterns during preimplantation development (Table S8). We found that from mature oocyte to two-cell stage embryo, the process that maternal transcripts are depleted and zygotic gene expression started, the genes that show more than 2 fold changes are clearly enriched for the basic cell metabolism related GO terms such as protein transport ($p<1.0X10^{-20}$), transcription ($p<1.0X10^{-20}$), regulation of transcription ($p<1.0X10^{-20}$), cell cycle ($p<9.9X10^{-13}$), modification-dependent protein catabolic process ($p<2.8X10^{-10}$), translation ($p<6.1X10^{-9}$), mRNA processing ($p<1.6X10^{-7}$), cell division ($p<2.0X10^{-7}$). This suggested that during the development period from shutdown of the maternal transcriptome to turn on the zygotic transcriptome, the general cell metabolism is thoroughly changed.

From two-cell to four-cell stage, the genes that changed their expression still show strong enrichment of general metabolism related GO terms (Table S8). Translation ($p<1.0X10^{-20}$), protein transport ($p<3.3X10^{-13}$), electron transport chain ($p<2.3X10^{-7}$), regulation of apoptosis ($p<2.9X10^{-7}$), tRNA processing ($p<8.3X10^{-6}$), and cell migration ($p<1.5X10^{-5}$) related GO terms are clearly enriched, indicating that the general metabolism is still reshaping from two-cell to four-cell stage embryos, probably because the delay of the translation of zygotic transcribed genes into significant amount of proteins to play the physiological functions.

From four-cell to eight-cell stage, the genes that changed their expression shifted to development and cell differentiation related GO terms (Table S8). For example, multicellular organismal development ($p<1.2X10^{-8}$), cell adhesion ($p<1.8X10^{-8}$), axon guidance ($p<4.0X10^{-6}$), cell differentiation ($p<2.1X10^{-5}$), regulation of small GTPase mediated signal transduction ($p<4.7X10^{-5}$), response to oxidative stress ($p<6.0X10^{-5}$), small GTPase mediated signal transduction ($p<6.1X10^{-5}$), cell surface receptor linked

signal transduction ($9.7X10^{-5}$) were consistently enriched. This is also compatible with the fact that, after the four-cell stage, the totipotency of the blastomeres is lost at eight-cell stage, which already differentiated compared to four-cell blastomeres.

From eight-cell to blastocyst stage, for the genes that changed their expression, they were enriched for the metabolism related GO terms again, compatible with the fact that with the compaction, formation of the blastocyst cavity, and other dramatic morphological changes, the metabolism of the blastomere cells also strongly shifted accordingly.

From E3.5 ICM to E4.5 Epiblast, the genes that changed their expression were mainly enriched of the metabolic process ($p<1.7X10^{-7}$), lipid biosynthetic process ($p<2.1X10^{-6}$), modification-dependent protein catabolic process ($p<1.5X10^{-5}$), protein amino acid phosphorylation ($p<5.7X10^{-5}$), ubiquitin-dependent protein catabolic process ($p<7.8X10^{-5}$). Interestingly, apoptosis ($p<9.0X10^{-6}$) is also enriched. This is compatible with the fact that, at this stage, there are significant apoptosis of mis-positioned cells in the blastocyst. Moreover, one day later, at E5.5 significant apoptosis happens in the epiblast to change them from epithelium into an egg cylinder with a cavity inside.

Alternative 3'UTRs during preimplantation development

To further understand the biological relevance of the global change of alternative 3’UTRs, we did Gene Ontology (GO) analysis for the genes that changed the length of their 3’UTR during preimplantation. From oocyte to two-cell stage, we found that the genes changing 3’UTR length not only showed enrichment for general metabolism related terms as expected, they also clearly enriched for development and signaling related GO terms, such as multicellular organismal development ($p<3.6X10^{-7}$), embryonic limb morphogenesis ($p<1.9X10^{-5}$), embryonic skeletal system morphogenesis ($p<3.8X10^{-5}$), anterior/posterior pattern formation ($p<4.3X10^{-5}$), and transmembrane receptor protein tyrosine kinase signaling pathway ($p<6.2X10^{-5}$). In other words, from oocyte to two-cell stage, although the genes changing their mRNA abundance did not show enrichment of development terms, the genes not changing mRNA abundance but changing 3’UTR length enriched for development and signaling related terms, which indicates that

potentially these latter class of genes changing their translational regulation and corresponding protein abundance. This is compatible with the notion that during maternal-zygotic transition, not only the general cell metabolism, but also the development and signaling program shows dramatic changes.

Then we looked at the relationship between the length of 3'UTRs and the expression dynamics. We found that during preimplantation development, the upregulated transcripts tend to increase their 3'UTR length. In fact, from oocyte to two-cell stage, 25.6% (1,604 out of 6,252) of the upregulated genes increased their 3'UTR length whereas only 1.2% (73 out of 6,252) decreased their 3'UTR length. This is not due to upregulated transcripts having more reads and better sequencing coverage, because from ICM to Epiblast, 9.5% (252 out of 2,666) of the upregulated genes lengthened their 3'UTRs whereas 8.0% (213 out of 2,666) of the upregulated genes shortened their 3'UTRs. On the contrary, during preimplantation development the downregulated genes tend to decrease their 3'UTR length. From two-cell to four-cell stage, 22.2% (1,052 out of 4,732) of them shortened their 3'UTRs whereas only 1.4% (66 out of 4,732) of them lengthened their 3'UTRs. Similarly this is not all due to downregulated transcripts having less reads and worse sequencing coverage, because from oocyte to two-cell, for the downregulated genes, 4.2% (85 out of 2,016) of them shortened their 3'UTR whereas 4.0% (80 out of 2,016) of them lengthened their 3'UTR.

It has been proposed that the lengthening of 3'UTR is due to alternative polyadenylation. We looked at the binding site for the CPSF polyadenylation factor, AAUAAA, which presents about 10 to 30 nucleotides upstream of polyadenylation cleavage sites. For transcripts with at least 2 RPM in a given cell type, we predict the polyadenylation cleavage site as the most 3' base, of the UCSC RefSeq database 3'UTR sequence, covered by at least 2 reads. No predictions are made for the 2,609 transcripts that missed 3'UTR sequence in UCSC database. If the predicted polyadenylation cleavage site is within 50nt from the annotated one, we call that annotated end as observed in our data. Out of the 26,154 RefSeq transcripts contained in UCSC database, we observed the same 3'UTR end for 15,926 transcripts in at least one cell type. From the total number of

transcripts, 15,210 (or 58.2%) transcripts have a CPSF binding motif sequences (AAUAAA) within 50nt upstream of their polyadenylation cleavage sites, 10,428 (or 68.5%) of such transcripts being observed in our data. From the remaining 10,944 transcripts that do not have a CPSF binding motif within 50nt upstream of their polyadenylation cleavage site, the same 3'UTR ends were observed for 5,498 transcripts (or 50.2%) suggesting that is more likely to observe the same polyadenylation cleavage site as in UCSC database, if the transcript contains a CPSF motif at its 3' end. In addition, we observed 2,579 transcripts that have a 3'UTR end at least 200nt shorter than the annotated one (potential proximal 3'UTR) which have a CPSF binding motif within 50nt, in at least one cell type. 1,153 such transcripts don't have a CPSF binding motif at the annotated 3' end, while 1,426 transcripts also have a CPSF binding motif at the annotated 3' end.

Expression dynamics of epigenetic regulators

To understand the contribution of epigenetic regulation to the preimplantation development, we checked the expression pattern of 114 known epigenetic regulators within individual cells during this period (Fig. S14). For these regulators, 113 were expressed in at least one stage during preimplantation development (except *Act16b*) and 99 of them show differential expression at different stages of development (FC >2 or <0.5, p<0.01). This is compatible with the fact that the epigenetic status of the genome is continuously remodeling during the preimplantation development.

We found that the oocyte expresses high levels of *Ezh1, Cbx1, Cbx2, Myst2, Setd1a, Arid1a*, and *Kdm6b* (Fig. S14). The expression of these genes decreased at the two-cell stage. The epigenetic regulators expressed at the highest level at the two-cell stage include repressive ones such as *Setdb1* (also known as *Eset*), *Suz12, Suv39h2, Hdac9, Bmi1, Prdm2, Rnf2, Npm2, Kdm1b, Kdm6a, Arid1b, Rps6ka5, Mll3, Nsd1,* and *Clock* (Fig. S14). The genes upregulated at the four-cell stage include repressive ones such as *Ehmt2* (also known as *G9*a), *Cbx5, Mbd1, Mbd3, Setd8, Hdac1, Hdac6, Suv420h1* (Fig. S14). And also *Nasp, Mybbp1a, Taf1, Kdm5a, Smarcc1, Smarca4, Smarcd2, Prmt5, Kdm3b,*

Kat2a. This indicates that the epigenetic regulation is intrinsically involved in preimplantation embryonic development.

First cell lineage determination event

The first cell lineages are established at E3.5 when the outer cells of the embryo become the trophectoderm, which later will form the placenta, and the inner cells become the pluripotent inner cell mass (ICM), which later will form the whole embryo proper. It is known that the trophectoderm forms tight junction and adheren junction to seal the blastocyst cavity, expressing specific transcription factors such as Cdx2, Eomes, Gata3, and specific intermediate filaments such as Keratin 7, Keratin8, and Keratin18. There is also expression of some specific epigenetic regulators, such as Dnmt3b and Dnmt3l. By contrast, ICM specifically expresses pluripotency genes, such as Oct4, Sox2, and Nanog. From our single cell RNA-Seq, we recovered essentially all known marker genes that are specifically expressed in TE or ICM (Fig. S15). Indeed, we found that 2,054 transcripts are enriched in TE compared with ICM, whereas 2,502 transcripts are enriched in ICM compared with TE. This shows that when the blastomeres segregate into ICM and TE, the gene expression between them is so dramatically different that 27.7% of expressed transcripts have already shown clear differential expression between these two lineages. When we looked at the GO terms for these 4,556 differentially expressed transcripts between ICM and TE, we found that the metabolism related GO terms are strongly enriched in them, such as metabolic process ($p<2.3X10^{-8}$), carbohydrate metabolic process ($p<2.6X10^{-8}$), steroid metabolic process ($p<1.5X10^{-5}$), cholesterol metabolic process ($p<1.8X10^{-5}$), and lipid metabolic process ($p<3.8X10^{-5}$). This is compatible with the fact that TE is differentiating and will incorporate to form the placenta whereas the ICM will develop to form the embryo proper. We also found that cell cycle ($p<6.1X10^{-6}$) and regulation of cell shape ($p<3.0X10^{-5}$) are enriched in the differentially expressed genes between ICM and TE. This is compatible with the dramatic difference of cell shape and cell cycle regulation between ICM and TE.

To dissect the gene network underlying pluripotency, we compared our data with the known gene network for Oct4 associated with pluripotency of embryonic stem cells. We

found that 43 out of the 45 Oct4 network genes expressed within the same individual ICM cell. Furthermore, we found that 20 out of these 45 Oct4 interacting genes show differential expression between TE and pluripotent ICM (Fig. S16). This shows that through counting RNA molecules in individual cells, we quantitatively captured a large proportion of Oct4 interacting genes connected to pluripotency.

Moreover, we also compared our data with the gene network for the pluripotency of the human embryonic stem cells. We found that 38 of them show differential expression between ICM and TE, indicating that at least part of the core regulatory network for pluripotency is conserved between mouse and human. Moreover, we found that for the organ morphology gene network, 31 out of 33 genes show clear differential expression between ICM and TE, and for cellular development, amino acid, molecular transport gene network, 31 out of 33 genes show differential expression between these two lineages. This indicates that more than the general metabolism differences, there are also clear developmental program differences between ICM and TE lineages.

Gene regulators related to pluripotency and reprogramming

Recently, it has been shown that Klf family genes are crucial during reprogramming of somatic cells to pluripotent iPS cells. We looked at the endogenously expressed Klf genes during early embryonic development (Fig. S17). We found that *Klf17* is the most abundantly expressed *Klf* gene during preimplantation development. It is highly expressed from oocyte to eight-cell stage. Then it is maintained in TE but strongly downregulated in ICM and epiblast, indicating that it is potentially important for the TE lineage. Similarly, *Klf16* is expressed at oocyte and two-cell stage, upregulated at four-cell and eight-cell stage, maintained in TE, but clearly decreased in ICM. *Klf7* expression is high in oocyte, decreased 3.2 fold at the two-cell stage, strongly decreased 23.2 fold at the four-cell stage, and kept at a very low level later. *Klf4*, *Klf10*, and *Klf11* are upregulated at the two-cell stage, and maintain their expression during later development. Interestingly, *Klf4* shows high expression in ICM, is downregulated in ES cells and shows very heterogeneous expression in ES cells, which may contribute to the intrinsic subpopulations of ES cells. *Klf5*, *Klf8*, and *Klf9* are upregulated from oocyte to two-cell

stage, strongly upregulated again at the four-cell stage, and maintain high expression at later stages (Fig. S17). *Klf2* expression is high in oocyte, downregulated at the two-cell stage, strongly upregulated at the four-cell stage and maintains high level at later stages. *Klf2*, *Klf3*, *Klf5*, *Klf8*, *Klf9*, *Klf10*, *Klf11*, and *Klf16* also show highly variable expression in ES cells. When compared ICM with TE, we found that *Klf6*, *Klf16*, *Klf17* are enriched in TE, whereas *Klf4* and *Klf8* are enriched in ICM. In ES cells, the most abundant Klf genes are *Klf2*, *Klf3*, *Klf6*, *Klf9*, and *Klf10*.

Then we looked at the Sox family genes, another family of genes important for reprogramming (Fig. S17). We found that *Sox2* is upregulated at the two-cell stage (14 fold), maintained at a significant level in the TE lineage, and strongly upregulated in the pluripotent ICM (11 fold), which is compatible with the fact that *Sox2* is both important for the maintenance of pluripotency and re-establishment of pluripotency during reprogramming. In fact, *Sox2* is the most abundant Sox gene expressed in preimplantation embryos. *Sox12* and *Sox13* expression is low at the two-cell stage, strongly upregulated at the four-cell stage (38 – 50 fold), and maintains high expression at later developmental stages. The expression of *Sox17* is clearly upregulated at the four-cell stage (5 fold), maintains similar levels in TE, but is strongly upregulated in ICM. *Sox15* is also strongly upregulated at the four-cell stage (83 fold), maintained at a high level in TE lineage, but is decreased in ICM and epiblast (3.3 fold). We also found that the expression of *Sox4*, *Sox11*, *Sox13*, and *Sox15* are very heterogeneous in ES cells, whereas that of *Sox2* and *Sox17* are quite consistent (Fig. S17).

Next we looked at the expression of *Myc* and *Lin28* (Fig. S17). *C-myc* is strongly upregulated at the two-cell (21.1 fold) and four-cell stage (28.3 fold) and maintains high expression at later stages. *N-myc* starts expression at the two-cell stage, is upregulated at the four-cell stage (3.1 fold), and further upregulated in epiblast. *L-myc* is expressed at low levels from oocyte to eight-cell stage, and is upregulated in the ICM. *N-myc* is more expressed than *Myc* and *L-myc* in ES cells. This is compatible with the fact that C-myc knockout ES cells maintain pluripotency, probably because of redundant function of *N-myc*. Lin28 is consistently expressed from oocyte to eight-cell stage, downregulated in

ICM, but upregulated later in epiblast. *Lin28b* also shows high expression throughout the preimplantation development.

We also looked at other reprogramming key factors, *Oct4*, *Nr5a2*, and *Nanog* (Fig. S17). *Oct4* is consistently expressed from oocyte to eight-cell stage, then maintains in the TE, but is strongly upregulated in ICM (6 fold), downregulated in epiblast, and shows some variability in ES cells. *Nanog* is not maternally expressed. It starts to express at the four-cell stage and is highly upregulated in the ICM (5.8 fold) and shows significant variable expression in ES cells. This indicates that, to counteract the programme in the eight-cell blastomeres for trophectoderm lineage, the ICM enhances the expression of the pluripotency master genes *Oct4*, *Sox2*, and *Nanog* to maintain and stabilize their pluripotency. *Nr5a2* is expressed in the oocyte, upregulated at the two-cell stage (4.5 fold), and further upregulated at the four-cell stage (6.2 fold). Then its high expression is maintained in TE, but decreased in ICM and epiblast (4.3 fold). It also shows highly heterogeneous expression in ES cells.

Gene regulators related to small RNA pathway

Small RNA pathways including microRNA, endo-siRNA, and piRNA pathway have been shown to be potentially crucial for early embryonic development. We looked at the expression dynamics of the biogenesis members of this pathway. We found that *Dicer* is expressed at very high levels in the mature oocyte, and gradually downregulated from the two-cell stage, and upregulated in E4.5 epiblast again. For *Dgcr8*, *Ago1*, and *Ago2*, they have low expression in mature oocytes, are strongly upregulated at the two-cell stage, further upregulated at the four-cell stage, and maintained high expression at later stages. *Ago4* is not expressed in oocyte, upregulated at the two-cell stage, clearly upregulated further at the four-cell stage, maintained at the eight-cell stage and TE lineage, but nearly lost in ICM and epiblast. *Ago3* is expressed at very high levels in oocyte and two-cell blastomeres. It is downregulated at the four-cell stage, and further downregulated in ICM and epiblast. Similarly, *Miwi* is highly expressed in mature oocyte and two-cell embryos, but nearly disappears after the four-cell stage. On the contrary, *Mili* is not present in oocytes. It is upregulated at the two-cell stage, further upregulated at four-cell stage, but

downregulated in epiblast. *Miwi2* is not expressed during preimplantation development. The high expression of Mili and Miwi points to the potential importance of maternal inheritance of piRNAs for preimplantation development. Interestingly, in ES cells, the most abundant Ago family members are *Ago1* and *Ago2*, whereas in mature oocytes, the most abundant member is *Ago3*.

SUPPLEMENTARY MATERIALS AND METHODS

False positives estimates

There are two sources of false positives for this method: (1) homozygous loci predicted as heterozygous, which can produce false ASE calls (Fig. S7); and (2) balanced allelic expression (heterozygous) loci which can also be falsely called as ASE (Fig. S6). The false positive rate produced by the first step is a function of the percentile used to detect homozygous loci (in our example the 95 percentile). It can be estimated as follows: if the search space contains n loci, and m of them are called heterozygous (have a minor allele frequency above 0.066), then the expected number of homozygous loci (false positives) among the remaining m loci is $\frac{q}{1-q}(n-m)$, where q = 0.95 is the percentile chosen for the minor allele frequency of homozygous loci. A higher percentile will ensure a reduced false positive rate produced by this step.

The false positive rate produced by the second step is a function of the p-value cut-off used to call ASE loci. An estimation of the error rate is m*p, which enables us to control the false positive rate produced by this step. Therefore, an upper bound of the expected false positive rate produced by this method is $\frac{q}{1-q}(n-m)+m*p$. For cells used for this analysis, the expected false positive rate ranges between 5% and 12% (Table S3).

3' UTR size variations

Coverage files produced by aggregating cells at the same developmental stage are used to investigate 3' UTR size variation. The search space is limited to RefSeq transcripts available from the UCSC Genome Browser website. The goal is to detect mRNA isoforms with shorter 3' UTRs compared to the annotated one.

Our single cell RNA-Seq analysis relies on the use of poly(T) primer for the reverse transcription step and is shown to produce better coverage of the 3' end of the mRNAs. In fact, we show that after about 1.5 kb from mRNA's 3' end, the reads coverage dropped

linearly and nearly disappeared [1] (Fig. S9 and Fig. S10). In order to detect shortening of 3'UTRs, we developed an algorithm that takes advantage of the particularities of this protocol. The algorithm assumes that the expected coverage of a single isoform, when screening mRNAs from the 3' to 5' end, starts with a region up to 100 nt long with increased coverage (produced by the protocol's fragmentation step and the fact that poly(A) fragments are blocked from being sequenced), followed by a region up to 1.5 kb long with linear decreasing coverage. Predictions of 3'UTR ends (cleavage sites) are produced for each individual gene iteratively by building a list of potential 3'UTR ends from local maximum points of observed gene coverage. The list of potential 3'UTR ends is extended with a new local maximum location, if the new genomic location is at least 250 nt away from current stored locations and the observed coverage is significantly higher than one expected to be produced from a current list of potential 3'UTR ends.

We used this method to predict cleavage sites for each individual cell type. We checked concordance of the predicted 3'UTR ends across 9 cell types and found 11,304 consensus genes for five out of the nine cell types, out of which, 2,108 genes have both long (distal) and short (proximal) 3'UTR isoforms (Table S5). Simultaneously, we looked for presence of CPSF (the canonical polyadenylation signal) motif (AAUAAA) within 100 nt of the predicted 3'UTR end. The CPSF motif was found in 60% of all predictions (consistent with the number of times this motif is found in the surrounding 3'UTR ends of RefSeq database) and 40% of the time for proximal predictions when multiple 3'UTR ends are predicted. In the majority of cases, the CPSF motif was found 20 nt downstream from the predicted cleavage site as expected [2] (Fig. 6).

Additionally we used the coverage height of the predicted 3'UTR ends to measure the relative expression levels between different isoforms of the same gene. We observed a systematic increase of expression of long (distal) isoforms relative to the short (proximal) isoforms when both 3'UTR isoforms are present (Fig. S13).

Real-time PCR validation of single cell RNA-Seq data

For TaqMan real-time PCR, 1.0 ul of diluted cDNAs of 2-cell and 4-cell embryos was used for each 10 ul real-time PCR (1X PCR Universal Master Mix, 250 nM TaqMan probe, 900 nM of each primer; these are commercially available as ready to use assays, custom-plated in 384-plates, or TaqMan low Density Array cards by Applied Biosystems). All reactions were duplicated. The PCR was done as following using an AB7900 with 384-well plates: first, 95°C for 10min to activate the Taq polymerase; then 40 cycles of 95°C for 15sec and 60°C for 1min. To confirm the reliability of our observations on differentially expressed genes by our single cell RNA-Seq, we compared 97 genes which were detected by both real-time PCR (Ct <32) and RNA-Seq (>0.1 RPM) and found that their correlation coefficient is 0.89, validating the accuracy of our single cell RNA-Seq data (Fig. S11 and Table S6).

For verification of the allele specific expression (ASE), we designed allele specific real-time PCR assays with the 3' most nucleotide matching the SNP nucleotide in the forward primers (Fig. S12). The qPCR preferentially amplified the allele with the matching SNP nucleotide, leaving the other allele with lower amplification. We designed 26 pairs of assays for the loci showing ASE in early blastomeres and analyzed them in the single blastomeres from two-cell, four-cell, and eight-cell stage embryos. We found that 23 out of 26 assays match to the RNA-Seq ASE results with a Pearson correlation coefficient greater than 0.85 between the allele specific qPCR log2 (allelic ratio) and SOLiD log2 (allelic ratio) (Table S7). Here, the averaged Ct values and standard deviations were calculated from three technical repeats.

REFERENCES

[1] Tang F, Barbacioru C, Wang Y, Nordman E, Lee C, Xu N, Wang X, Bodeau J, Tuch BB, Siddiqui A, Lao K, Surani MA. mRNA-Seq whole-transcriptome analysis of a single cell. *Nat Methods* **6**, 377 (2009)

[2] Zhang K, Li JB, Gao Y, Egli D, Xie B, Deng J, Li Z, Lee JH, Aach J, Leproust EM, Eggan K, Church GM. Digital RNA allelotyping reveals tissue-specific and allele-specific gene expression in human. *Nat Methods* **6**, 613 (2009).

[3] Tang F, Barbacioru C, Bao S, Lee C, Nordman E, Wang X, Lao K, Surani MA. Tracing the derivation of embryonic stem cells from the inner cell mass by single-cell RNA-Seq analysis. *Cell Stem Cell* **6**, 468 (2010).

SUPPLEMENTARY FIGURE LEGENDS

Fig. S1. The potential underestimation or overestimation of ASE within individual cells based on bulk analysis. (A) The number of genes showing ASE in an individual cell will be underestimated based on the bulk amount of cells analyzed if the ASE only happens in a subset of cells within the population due to cell cycle, circadian clock, intrinsic heterogeneity, etc. And at least some of the genes showing allelic specific expression in subpopulations of cells will be missed and cannot be detected. (B) The number of genes showing ASE in an individual cell will be overestimated based on the bulk amount of cells analyzed if the ASE happens in different subsets of cells within the population due to cell cycle, circadian clock, intrinsic heterogeneity, etc. (C) The number of genes showing ASE in an individual cell will be underestimated based on the bulk amount of cells analyzed if the ASE happens at opposite direction in different subsets of cells within the population due to cell cycle, circadian clock, intrinsic heterogeneity, etc. And at least some of the genes showing allelic specific expression in subpopulations of cells will be missed and cannot be detected.

Fig. S2. Scatter plots of blastomeres of 2-cell, 4-cell and 8-cell stage embryos. The numbers in the boxes are the Pearson correlation coefficients of the corresponding scatter plots. The red lines indicate the 2-fold up/down expression changes. Data are listed in Table S1. Note: for each embryonic stage, blastomeres A1 & A2 are from the same embryo A, blastomeres B1 & B2 are from the same embryo B, and so on.

Fig. S3. Scatter plots of blastomeres of pre-implantation embryos. The numbers in the boxes are the Pearson correlation coefficients of the corresponding scatter plots. The red lines indicate the 2-fold up/down expression changes. Data is listed in Table S1.

Fig. S4. The schematic coverage plot showing the allelic specific expression (ASE) of a gene in two blastomeres from the same two-cell embryo. Data is listed in Table S2.

Fig. S5. Allelic specific gene expression calls in mouse ES cells. (A) The histograms of allelic ratios for genomic positions in ES cells. For a given genomic coordinate, the “allelic ratio” is the log2 of the number of reads aligned across that position, representing the reference nucleotide reads divided by the number of reads of the first non-reference nucleotide in dbSNP. If the alleles tend to be expressed at an equal level, we see a trimodal distribution of allelic ratios, representing the three possible genotypes: homozygous reference alleles, heterozygous alleles with equal expression of the two alleles, and homozygous alternative alleles. As expected, we mainly see reference and alternative homozygous alleles for the 12 nearly homozygous ES cells, and a few heterozygous alleles consisting of less than 0.3%, which are likely due to ES cells which were not 100% pure inbred. The aggregated ES cells #1 – #6 and ES cells #7 – #12 are shown in red and blue, respectively. **(B)** Distribution of the minor allele frequency in 12 mouse individual ES cells obtained from the same ES cell line, which is originally derived from a nearly homozygous embryo. The distribution of the allele ratio shows that there is a 95% chance for a homozygous locus (with at least 2 alleles observed; see SOM for details on loci selection) to generate a minor allele frequency of 0.066 or lower. The 4.7% of loci which have their minor allele frequency between 0.066 and 0.15 are potentially ASE loci, instrument errors, or mis-incorporations of bases during RT-PCR. The 0.3% of loci showing a minor allele frequency above 0.15 are likely to be ASE loci, due to less than 100% pure inbred ES cells, or potential imperfect gene duplications.

Fig. S6. Identification of balanced allele expression loci. The balanced allele expression loci of blastomeres from four 2-cell stage embryos are shown (A-H). Consider that we have a SNP with x copies of a1= 5' ATC**G**CCC 3' and y copies of a2 = 5' ATC**A**CCC 3'. The second strand is synthesized and x and y copies of the alleles b1 = 3' TAG**C**GGG 5' and b2 = 3' TAG**T**GGG 5' are complementarily produced. The ratio of (# a1 observations + # b2 observations) / (# a2 observations + # b1 observations) should be equal to 1 if no library of instrument biases exists, irrespective if the locus is allelic balanced or not. The distribution of this statistic generated from all heterozygous loci of an individual cell is represented here, and represents an approximation of the distribution of allelic ratios of balanced loci, taking in consideration library and instrument biases.

Fig. S7. Allele specific expression calls for blastomeres from four 2-cell stage embryos are shown (A-H). Locus is called to have allele specific expression (ASE) if the minor allele frequency is above 0.066 and the resulting p-value < 0.01 (generated from the distribution of allelic ratios of balanced loci). The distribution of 2-cell blastomeres with minor allele frequency above 50% is due to smoothing by the R script density plot.

Fig. S8. Heterozygous loci are sorted based on observed coverage. (A) Counts of allelic specific expression (ASE) loci as a function of coverage. There are significantly more ASE loci for the expressed genes, which have lower coverage. (B) The percentage of ASE out of all heterozygous loci (number of loci showing ASE)/(number of heterozygous loci) is relatively constant around 40% between 25-100x coverage. The large variations for 200x or more coverage are likely due to the stringencies in ASE detection. ASE calls are listed in Table 1.

Fig. S9. The 3'UTR coverage plots of Mycbp for mature oocyte, blastomeres in 2-cell, 4-cell and 8-cell stage embryos. The blastomeres of 2-cell stage embryos express both short (proximal) and long (distal) 3'UTRs equally while there are significantly more abundant expression of short 3'UTRs than the long 3'UTRs in other stages. The vertical bars at the top part of the plots indicate the predicted binding sites of miRNAs.

Fig. S10. 3' UTR prediction box plots of the long (distal) (A) and short (proximal) (B) isoforms. The mean lengths of the long 3'UTRs increase from mature oocyte to 4-cell and 8-cell stages, and are only slightly shorter than the lengths of 3'UTRs in RefSeq. In contrast, the mean lengths of the short 3'UTRs are significantly shorter than the lengths of 3'UTRs in RefSeq. For illustrative purposes, only the 3'UTRs with predictions shorter than 4,000 nt are used. The corresponding data is listed in Table S5. Publicly available data, GSE20187 [3], was added to data included in GSE22182 for this figure.

Fig. S11. TaqMan Real-time PCR validation of single cell RNA-Seq data. 2-cell vs. 4-cell. The correlation plot of fold changes determined by (A) TaqMan (real-time PCR) vs. SOLiD (RNA-Seq) and (B) the distributions of CV (standard deviation / mean).

Fig. S12. The allele specific real-time PCR validation of ASE detected in single cell RNA-Seq data. (A) The sketch of the assay design and (B) The representative allele specific real-time PCR curves. (C) The correlation plots of ASE detected by single cell RNA-Seq and allele specific real-time PCR.

Fig. S13. Alternative 3' UTRs during preimplantation development. Counts (A) and ratios (B) of transcripts showing short (proximal) 3'UTR isoforms (out of all RefSeq transcripts). The ratios of short vs. long 3'UTRs sizes decrease from mature oocyte to 4-cell and 8-cell stages, to 0.39 and further down to 0.35 in ESC. Publicly available data, GSE20187 [3], was added to data included in GSE22182 for this figure. (C) Box plots of the predicted ratio of proximal and distal 3'UTR for eight types of cells. We use the height of the predicted cleavage site to measure the relative expression levels of the isoforms. We observed a systematic increase of expression of long (distal) isoforms relative to the short (proximal) isoforms when both 3'UTR isoforms are present. Publicly available data, GSE20187 [3], was added to data included in GSE22182 for this figure. (D) The ratio of expression levels between the proximal (short) and distal (long) 3'UTR of the same genes during early embryonic development. The short 3'UTR isoforms have on average 6 times more expression than the corresponding long 3'UTR isoforms. The percentage of short 3'UTRs decreases in general during early embryonic development. The short 3'UTRs are about 5.3 times more abundant than the long 3'UTRs at the 2-cell stage. At 4-cell and 8-cell stages, the ratios of transcripts with short vs. long 3'UTRs increases to 6.3 – 6.9 fold. At the later development stages, the ratios decrease dramatically to 2.1 – 4.6 fold.

Fig. S14. Dynamic expression of epigenetic regulators within individual cells during preimplantation development. (A) The ones upregulated at 2-cell stage and maintained at a high level of expression thereafter. (B) Dynamic expression of Dnmt3a, 3b, 3l. (C) The

ones downregulated at 2-cell stage. (D) The ones upregulated at 2-cell stage and downregulated at 4-cell stage. (E) The ones upregulated at 4-cell stage and kept at a high level of expression thereafter.

Fig. S15. Differential expression of known marker genes for ICM and trophectoderm lineages.

Fig. S16. Gene Network Analysis of Oct4 in Embryonic Stem Cell Pluripotency Pathway using Ingenuity Systems software (www.ingenuity.com). The 22 genes (including Oct4) showing differential expression between ICM and trophectoderm were shown in red.

Fig. S17. Dynamic expression of pluripotency related genes. (A-B) Klf family genes within individual cells during preimplantation development. The ones upregulated at 4-cell stage. (C) Expression dynamics of Oct4, Nanog, and Nr5a2. (D-E) Sox family genes within individual cells during preimplantation development. The ones upregulated at 4-cell stage. (F) Myc and Lin28 family genes within individual cells during preimplantation development.

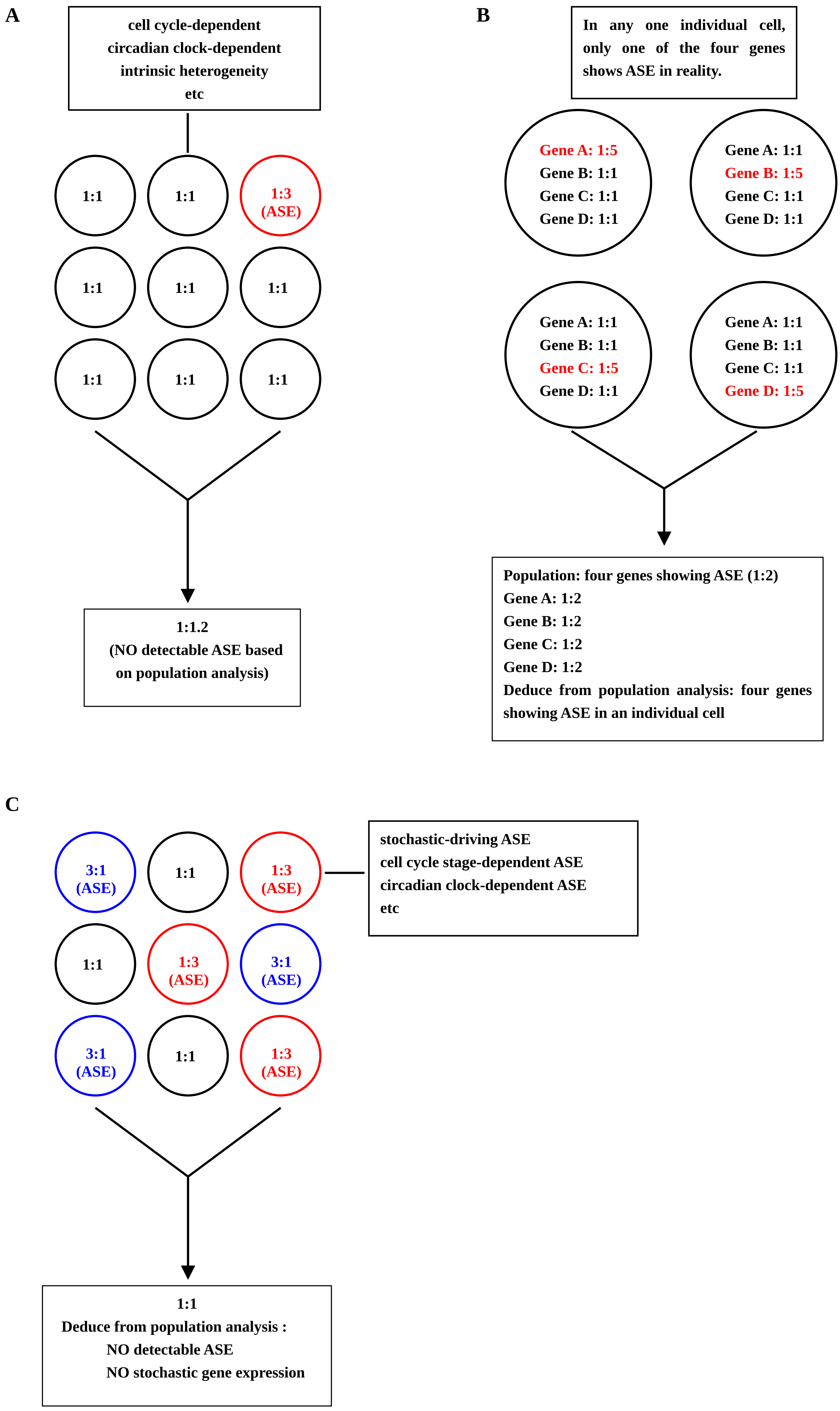
A
cell cycle-dependent
circadian clock-dependent
intrinsic heterogeneity
etc
1:1
1:1
1:3
(ASE)
1:1
1:1
1:1
1:1
1:1
1:1
1:1.2
(NO detectable ASE based on population analysis)
B
In any one individual cell, only one of the four genes shows ASE in reality.
Gene A: 1:5
Gene B: 1:1
Gene C: 1:1
Gene D: 1:1
Gene A: 1:1
Gene B: 1:5
Gene C: 1:1
Gene D: 1:1
Gene A: 1:1
Gene B: 1:1
Gene C: 1:5
Gene D: 1:1
Gene A: 1:1
Gene B: 1:1
Gene C: 1:1
Gene D: 1:5
Population: four genes showing ASE (1:2)
Gene A: 1:2
Gene B: 1:2
Gene C: 1:2
Gene D: 1:2
Deduce from population analysis: four genes showing ASE in an individual cell
C
3:1
(ASE)
1:1
1:3
(ASE)
1:1
1:3
(ASE)
3:1
(ASE)
3:1
(ASE)
1:1
1:3
(ASE)
stochastic-driving ASE
cell cycle stage-dependent ASE
circadian clock-dependent ASE
etc
1:1
Deduce from population analysis :
NO detectable ASE
NO stochastic gene expression

Figure S1

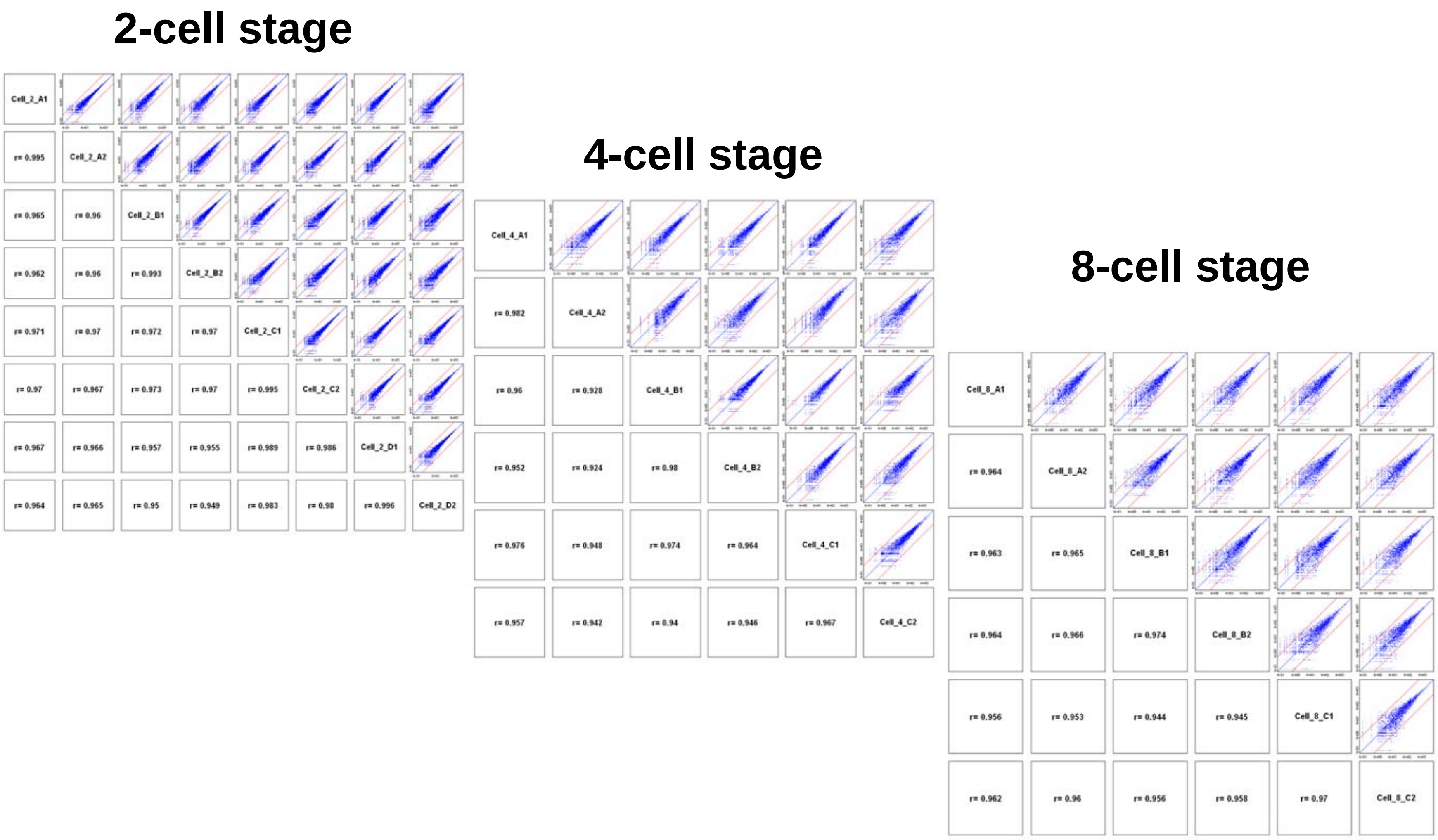
2-cell stage
Cell_2_A1
r= 0.995
Cell_2_A2
r= 0.965
r= 0.96
Cell_2_B1
r= 0.962
r= 0.96
r= 0.993
Cell_2_B2
r= 0.971
r= 0.97
r= 0.972
r= 0.97
Cell_2_C1
r= 0.97
r= 0.967
r= 0.973
r= 0.97
r= 0.995
Cell_2_C2
r= 0.967
r= 0.966
r= 0.957
r= 0.955
r= 0.989
r= 0.986
Cell_2_D1
r= 0.964
r= 0.965
r= 0.95
r= 0.949
r= 0.983
r= 0.98
r= 0.996
Cell_2_D2
4-cell stage
Cell_4_A1
r= 0.982
Cell_4_A2
r= 0.96
r= 0.928
Cell_4_B1
r= 0.952
r= 0.924
r= 0.98
Cell_4_B2
r= 0.976
r= 0.948
r= 0.974
r= 0.964
Cell_4_C1
r= 0.957
r= 0.942
r= 0.94
r= 0.946
r= 0.967
Cell_4_C2
8-cell stage
Cell_8_A1
r= 0.964
Cell_8_A2
r= 0.963
r= 0.965
Cell_8_B1
r= 0.964
r= 0.966
r= 0.974
Cell_8_B2
r= 0.956
r= 0.953
r= 0.944
r= 0.945
Cell_8_C1
r= 0.962
r= 0.96
r= 0.956
r= 0.958
r= 0.97
Cell_8_C2

Figure S2

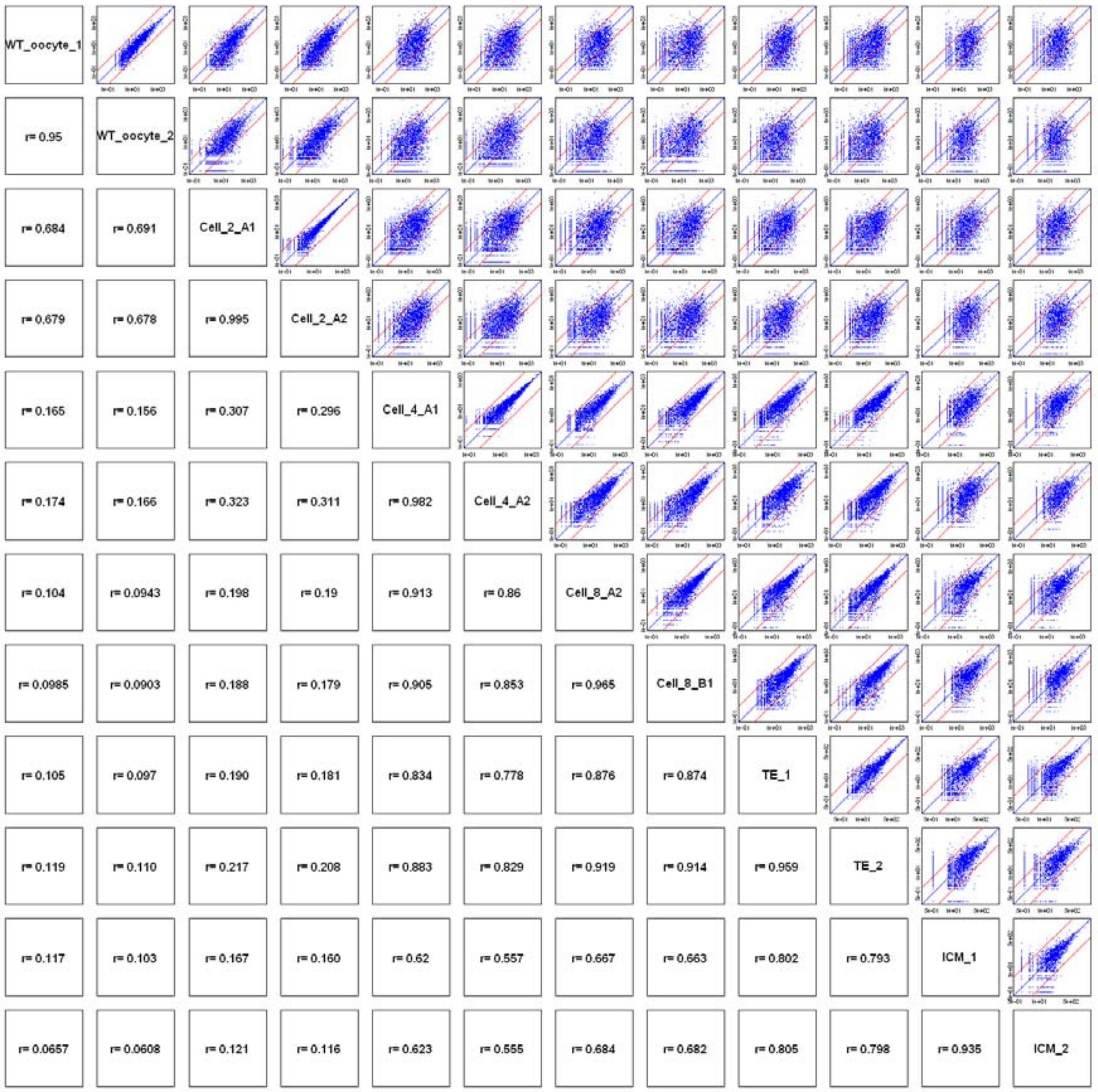
WT_oocyte_1
r= 0.95
WT_oocyte_2
r= 0.684
r= 0.691
Cell_2_A1
r= 0.679
r= 0.678
r= 0.995
Cell_2_A2
r= 0.165
r= 0.156
r= 0.307
r= 0.296
Cell_4_A1
r= 0.174
r= 0.166
r= 0.323
r= 0.311
r= 0.982
Cell_4_A2
r= 0.104
r= 0.0943
r= 0.198
r= 0.19
r= 0.913
r= 0.86
Cell_8_A2
r= 0.0985
r= 0.0903
r= 0.188
r= 0.179
r= 0.905
r= 0.853
r= 0.965
Cell_8_B1
r= 0.105
r= 0.097
r= 0.190
r= 0.181
r= 0.834
r= 0.778
r= 0.876
r= 0.874
TE_1
r= 0.119
r= 0.110
r= 0.217
r= 0.208
r= 0.883
r= 0.829
r= 0.919
r= 0.914
r= 0.959
TE_2
r= 0.117
r= 0.103
r= 0.167
r= 0.160
r= 0.62
r= 0.557
r= 0.667
r= 0.663
r= 0.802
r= 0.793
ICM_1
r= 0.0657
r= 0.0608
r= 0.121
r= 0.116
r= 0.623
r= 0.555
r= 0.684
r= 0.682
r= 0.805
r= 0.798
r= 0.935
ICM_2

Figure S3

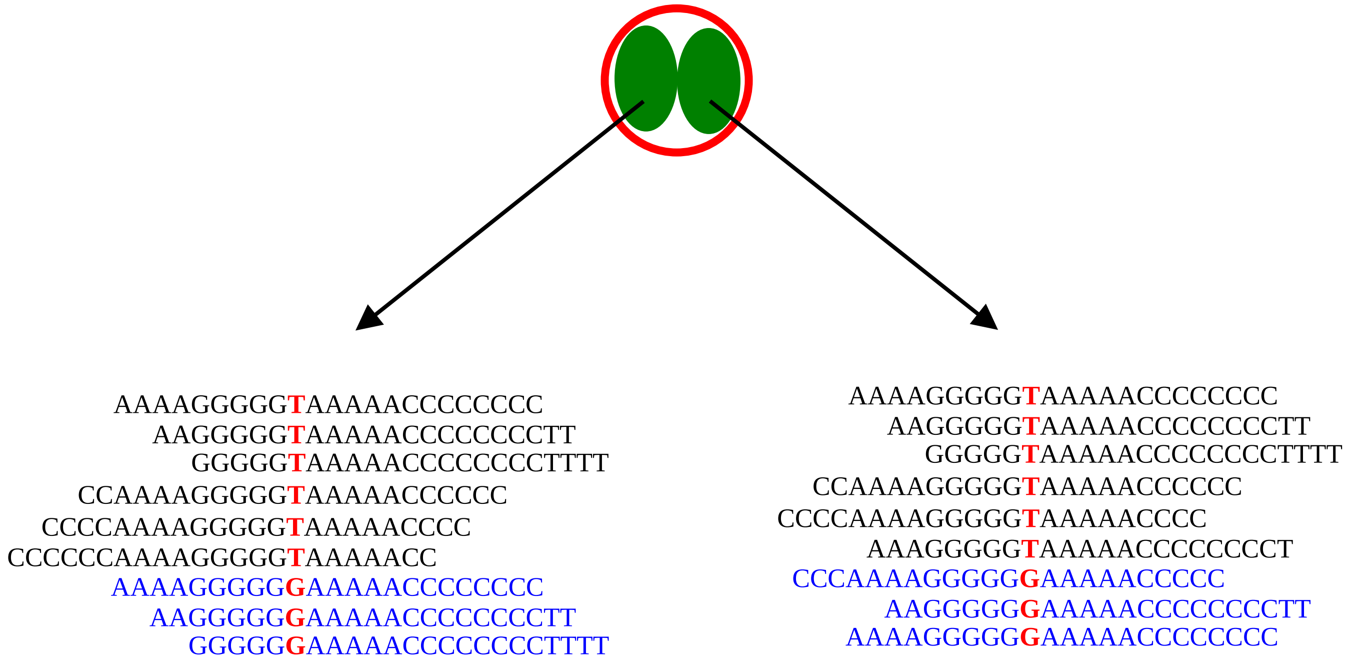

AAAAGGGGGTAAAAACCCCCCCC
AAGGGGGTAAAAACCCCCCCCTT
GGGGGTAAAAACCCCCCCCTTTT
CCAAAAGGGGGTAAAAACCCCCC
CCCCAAAAGGGGGTAAAAACCCC
CCCCCCAAAAGGGGGTAAAAACC
AAAAGGGGGGAAAAACCCCCCCC
AAGGGGGGAAAAACCCCCCCCTT
GGGGGGAAAAACCCCCCCCTTTT
AAAAGGGGGTAAAAACCCCCCCC
AAGGGGGTAAAAACCCCCCCCTT
GGGGGTAAAAACCCCCCCCTTTT
CCAAAAGGGGGTAAAAACCCCCC
CCCCAAAAGGGGGTAAAAACCCC
AAAGGGGGTAAAAACCCCCCCT
CCCAAAAGGGGGGAAAAACCCCC
AAGGGGGGAAAAACCCCCCCCTT
AAAAGGGGGGAAAAACCCCCCCC

Figure S4

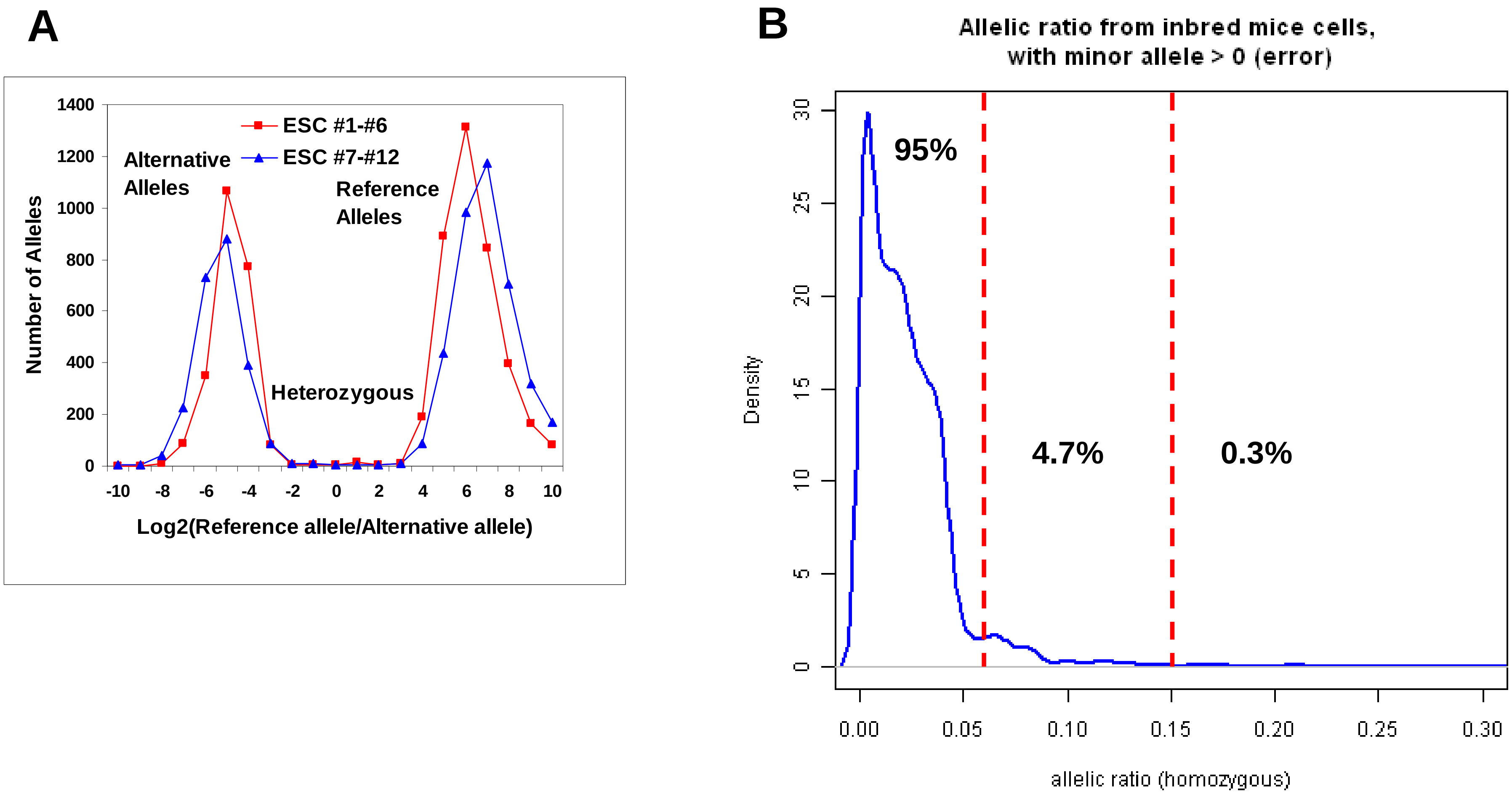
A
1400
1200
1000
800
600
400
200
0
Number of Alleles
ESC #1-#6
ESC #7-#12
Alternative
Alleles
Reference
Alleles
Heterozygous
-10
-8
-6
-4
-2
0
2
4
6
8
10
Log2(Reference allele/Alternative allele)
B
Allelic ratio from inbred mice cells,
with minor allele > 0 (error)
Density
30
25
20
15
10
5
0
95%
4.7%
0.3%
0.00
0.05
0.10
0.15
0.20
0.25
0.30
allelic ratio (homozygous)

Figure S5

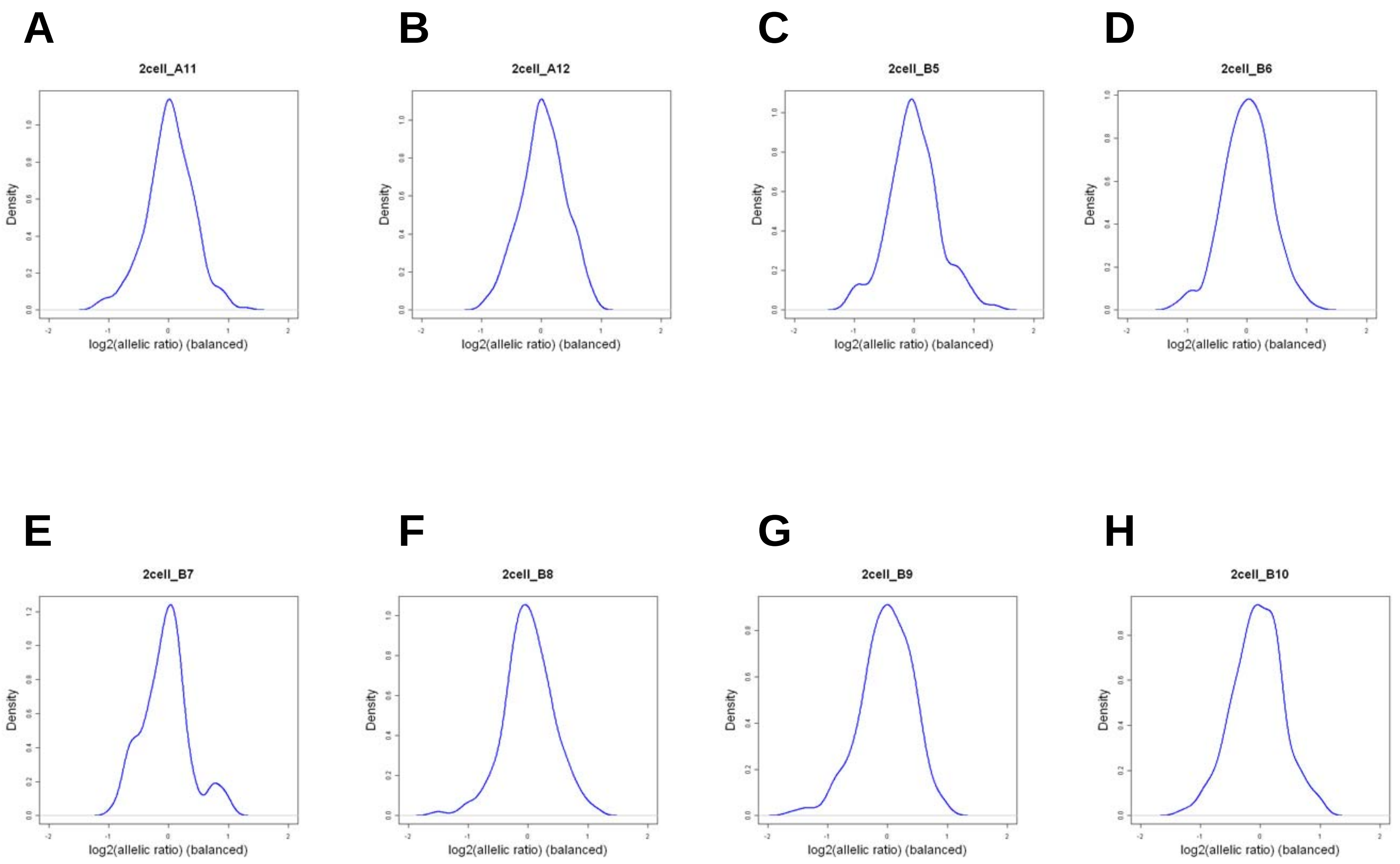

A
B
C
D
E
F
G
H
2cell_A11
2cell_A12
2cell_B5
2cell_B6
2cell_B7
2cell_B8
2cell_B9
2cell_B10
Density
log2(allelic ratio) (balanced)

Figure S6

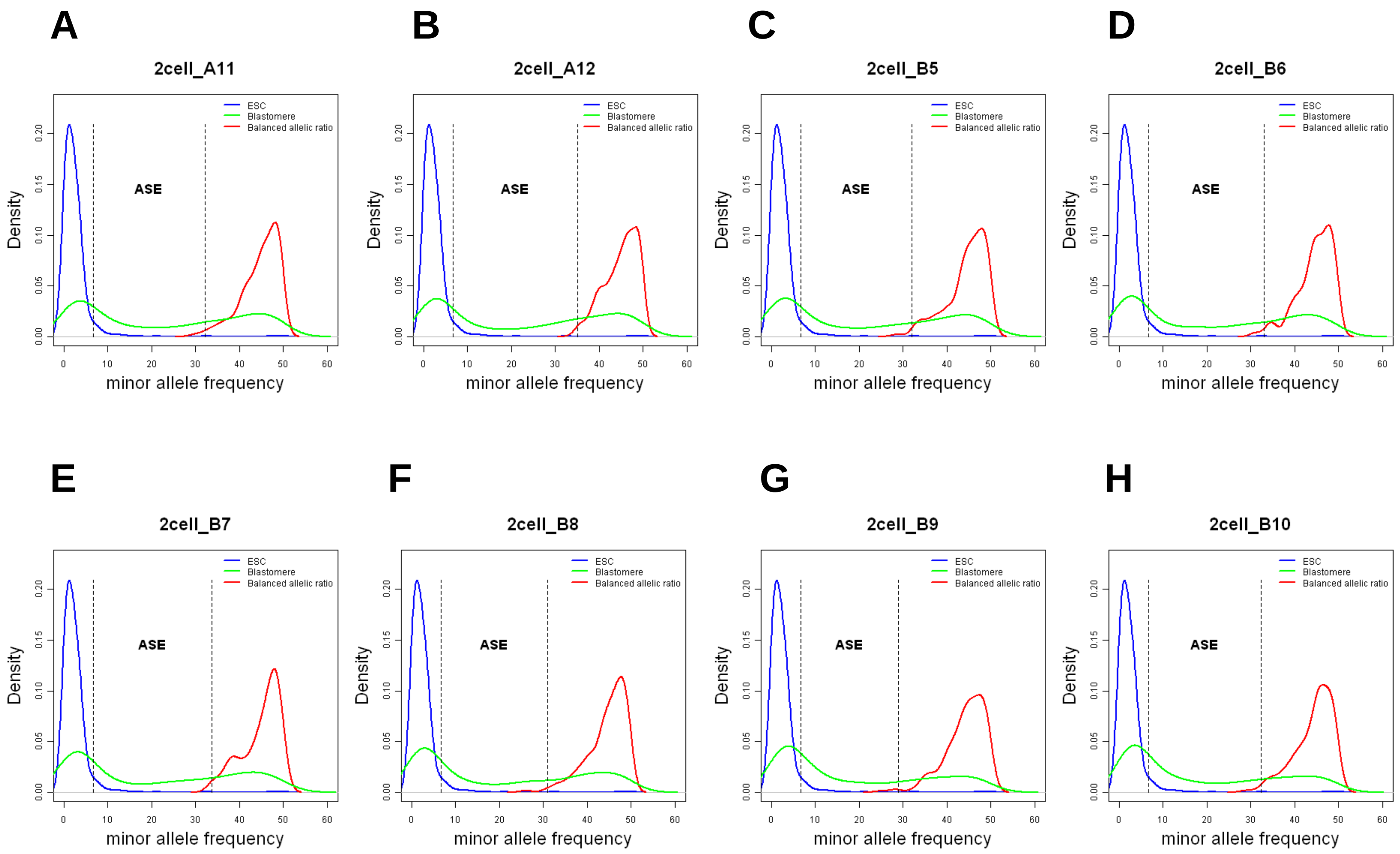
A
2cell_A11
B
2cell_A12
C
2cell_B5
D
2cell_B6
E
2cell_B7
F
2cell_B8
G
2cell_B9
H
2cell_B10
ESC
Blastomere
Balanced allelic ratio
ASE
Density
minor allele frequency
0.00
0.05
0.10
0.15
0.20
0
10
20
30
40
50
60

Figure S7

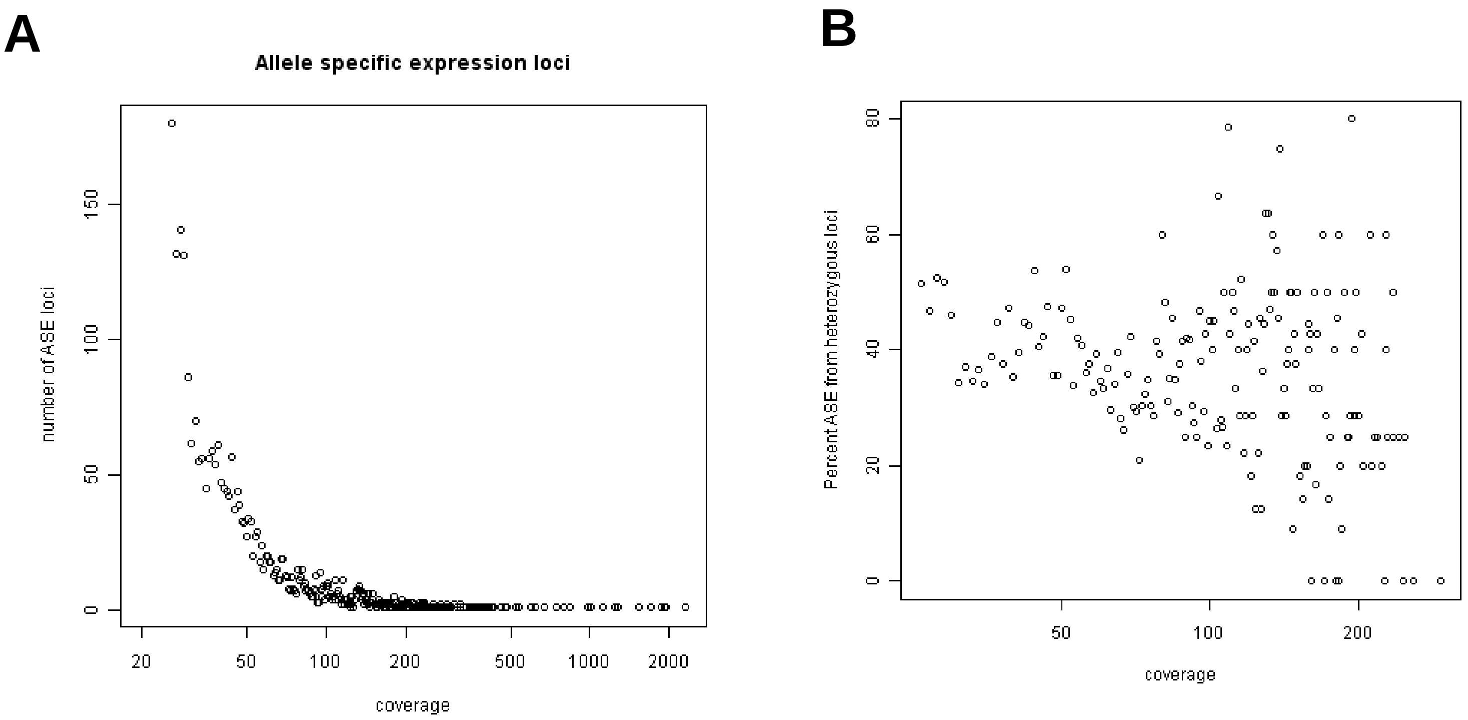
A
Allele specific expression loci
number of ASE loci
0
50
100
150
20
50
100
200
500
1000
2000
coverage
B
Percent ASE from heterozygous loci
0
20
40
60
80
50
100
200
coverage

Figure S8

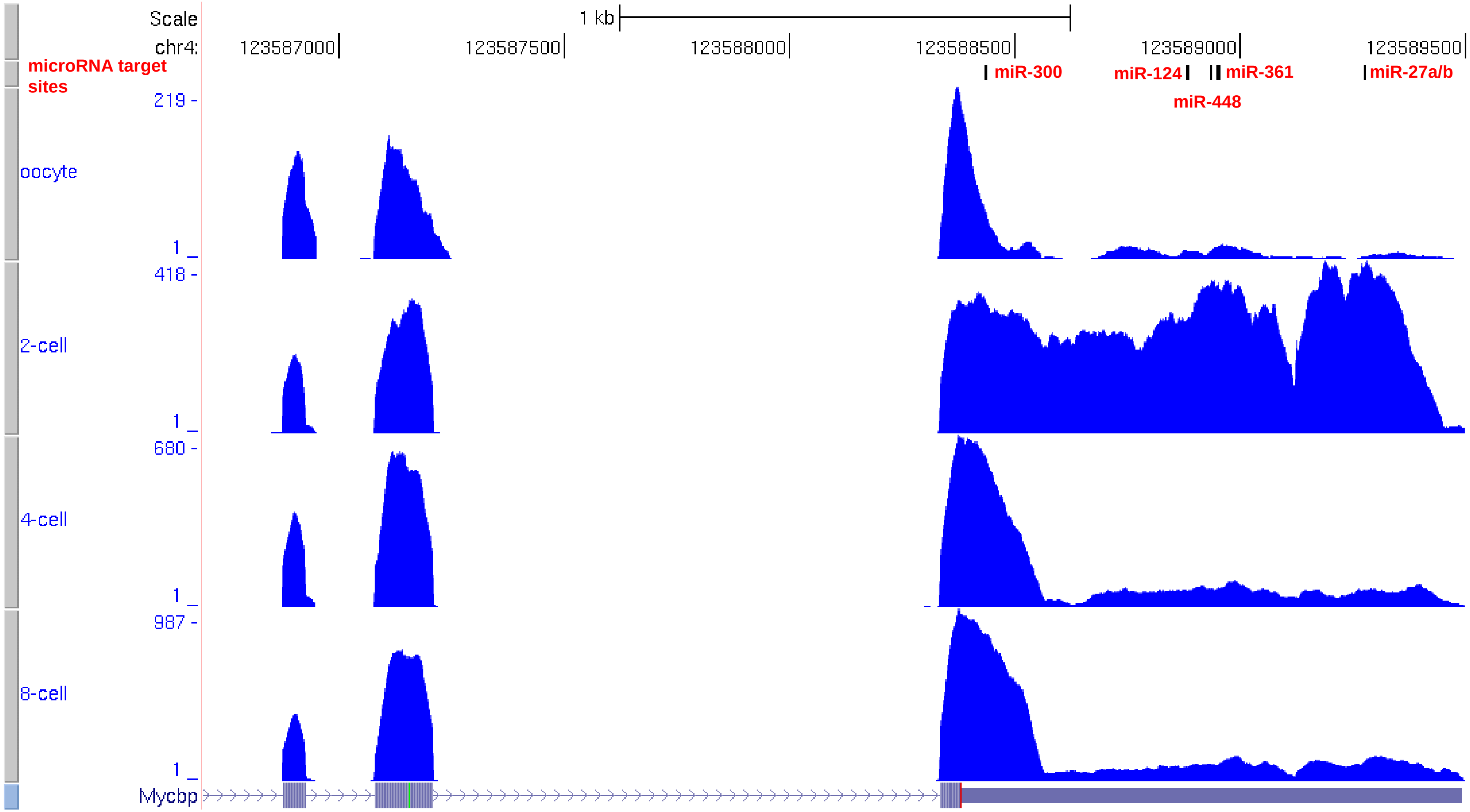
Scale
chr4:
1 kb
123587000
123587500
123588000
123588500
123589000
123589500
microRNA target sites
miR-300
miR-124
miR-361
miR-27a/b
miR-448
219 -
1 _
oocyte
418 -
1 _
2-cell
680 -
1 _
4-cell
987 -
1 _
8-cell
Mycbp

Figure S9

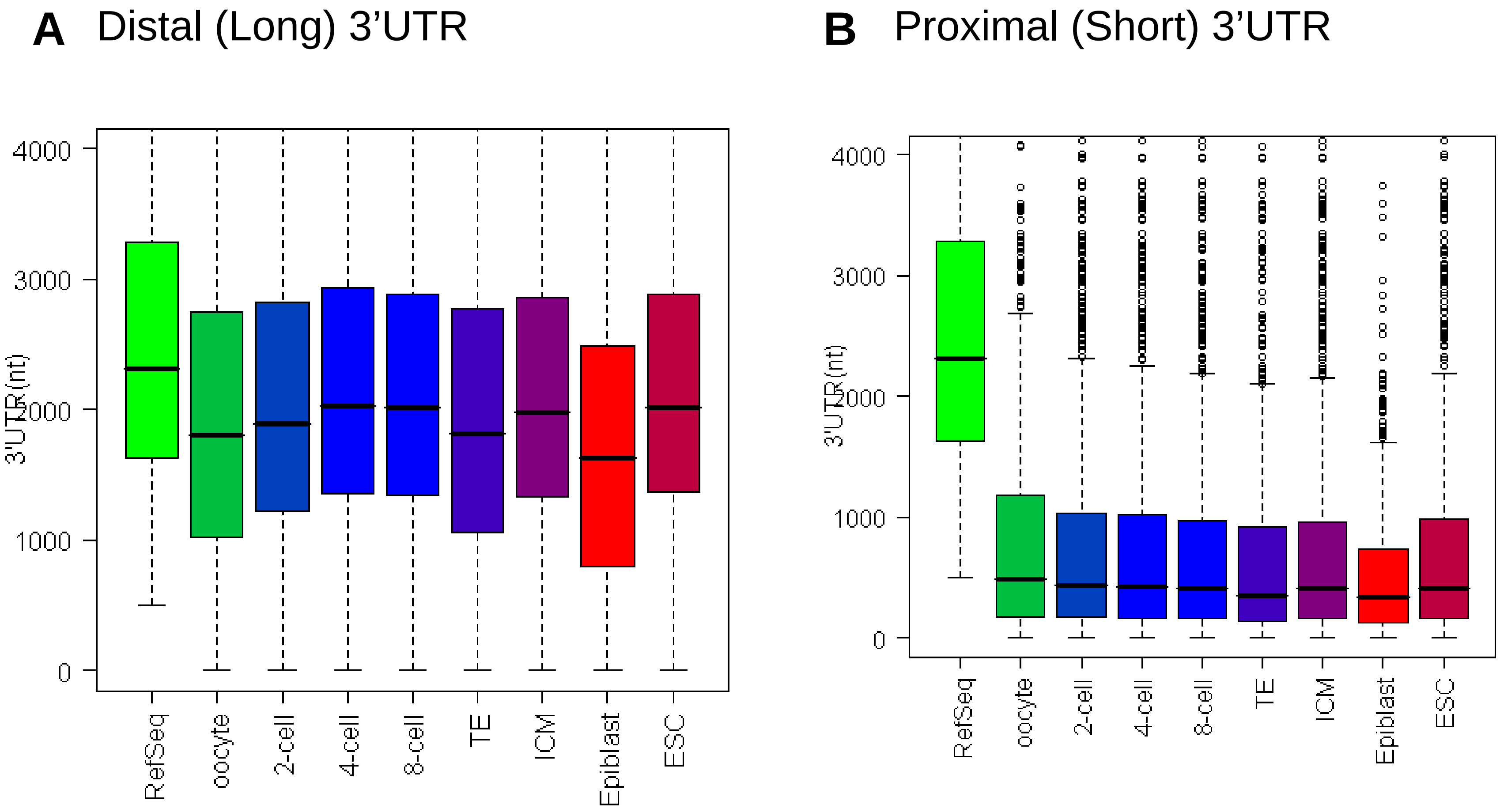

A Distal (Long) 3'UTR
B Proximal (Short) 3'UTR
3'UTR(nt)
0
1000
2000
3000
4000
RefSeq
oocyte
2-cell
4-cell
8-cell
TE
ICM
Epiblast
ESC

Figure S10

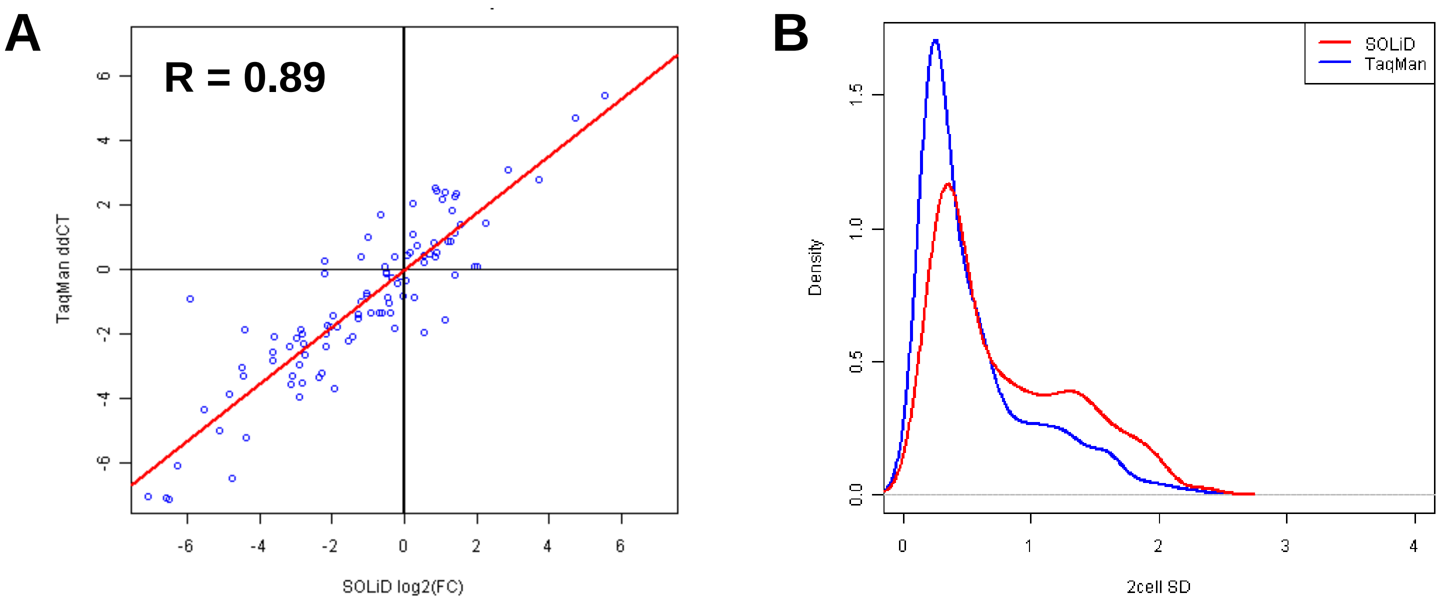

A
R = 0.89
TaqMan ddCT
SOLiD log2(FC)
B
SOLiD
TaqMan
Density
2cell SD

Figure S11

A **Allelic specific PCR:**

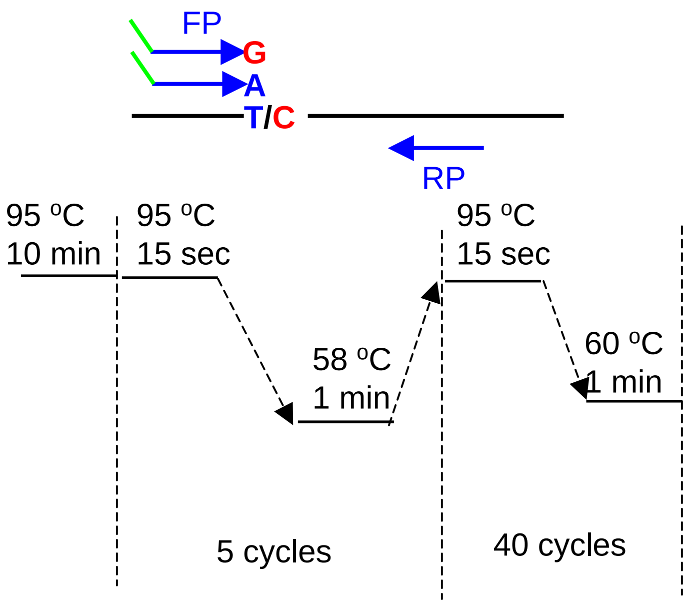

B **Representative allelic specific qPCR curves:**

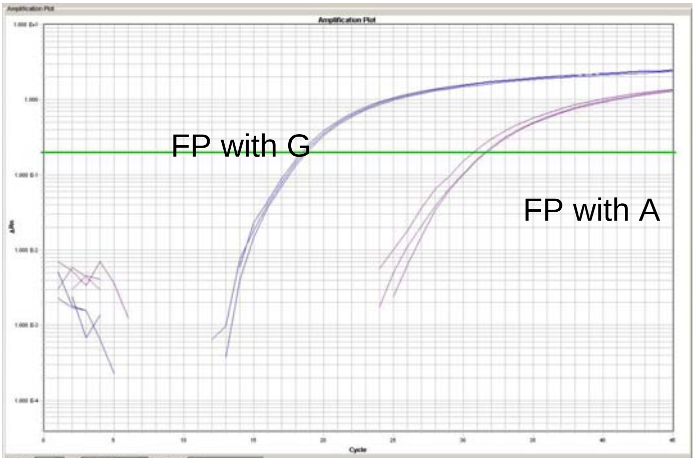

C

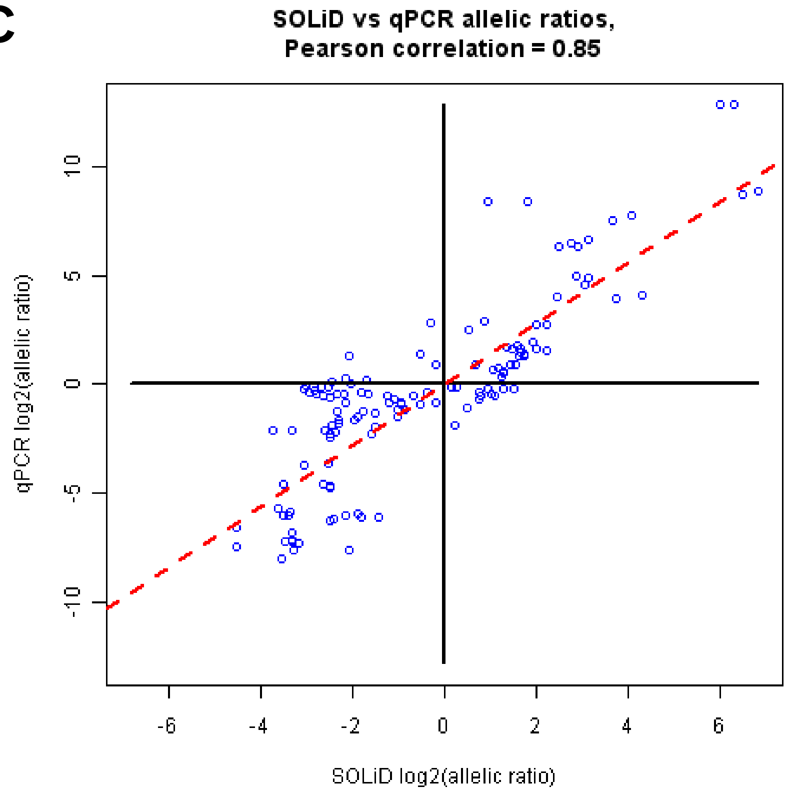

Figure S12

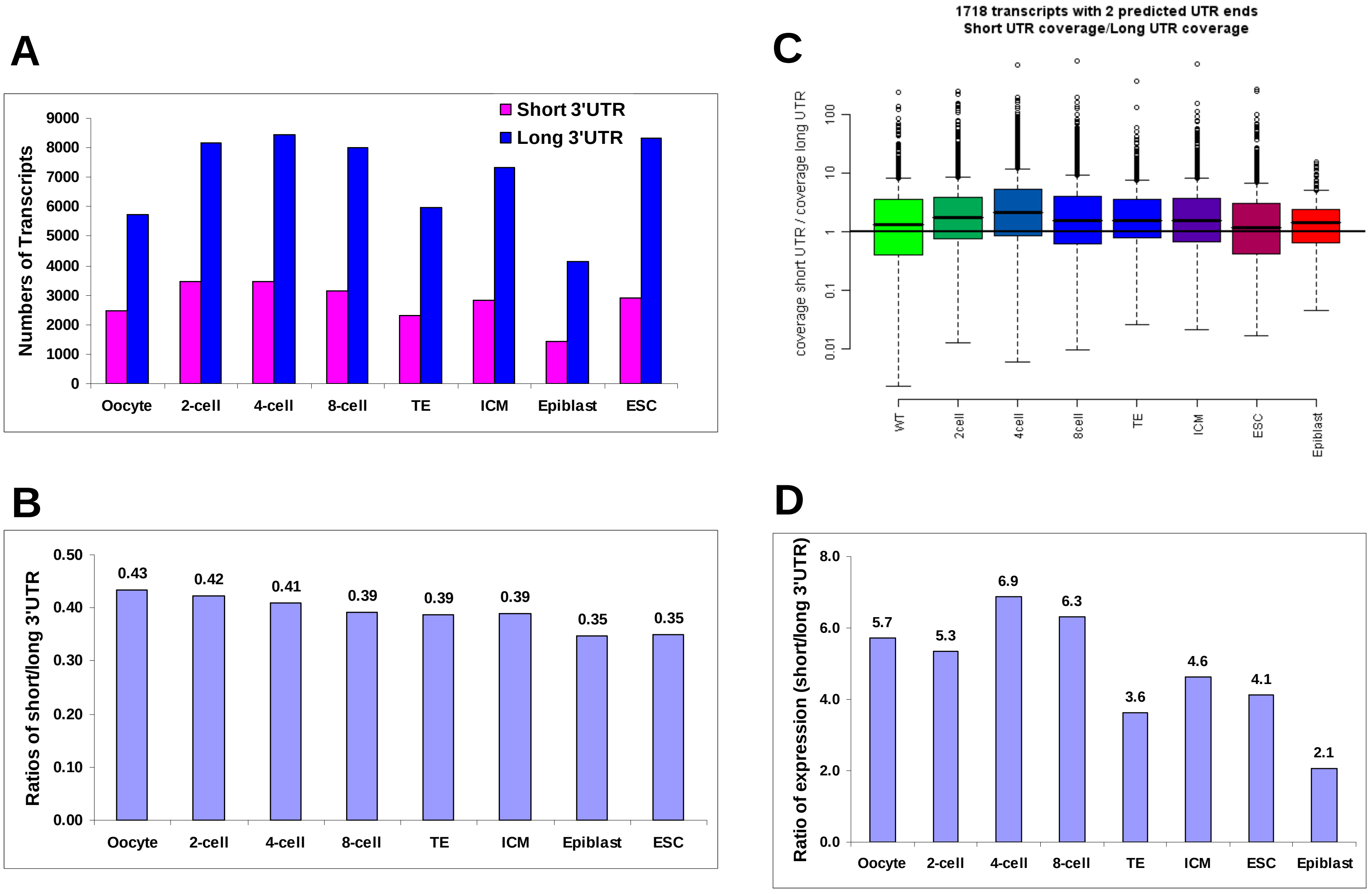
A
Short 3'UTR
Long 3'UTR
Numbers of Transcripts
0
1000
2000
3000
4000
5000
6000
7000
8000
9000
Oocyte
2-cell
4-cell
8-cell
TE
ICM
Epiblast
ESC
B
Ratios of short/long 3'UTR
0.00
0.10
0.20
0.30
0.40
0.50
0.43
0.42
0.41
0.39
0.39
0.39
0.35
0.35
Oocyte
2-cell
4-cell
8-cell
TE
ICM
Epiblast
ESC
C
1718 transcripts with 2 predicted UTR ends
Short UTR coverage/Long UTR coverage
coverage short UTR / coverage long UTR
0.01
0.1
1
10
100
WT
2cell
4cell
8cell
TE
ICM
ESC
Epiblast
D
Ratio of expression (short/long 3'UTR)
0.0
2.0
4.0
6.0
8.0
5.7
5.3
6.9
6.3
3.6
4.6
4.1
2.1
Oocyte
2-cell
4-cell
8-cell
TE
ICM
ESC
Epiblast

Figure S13

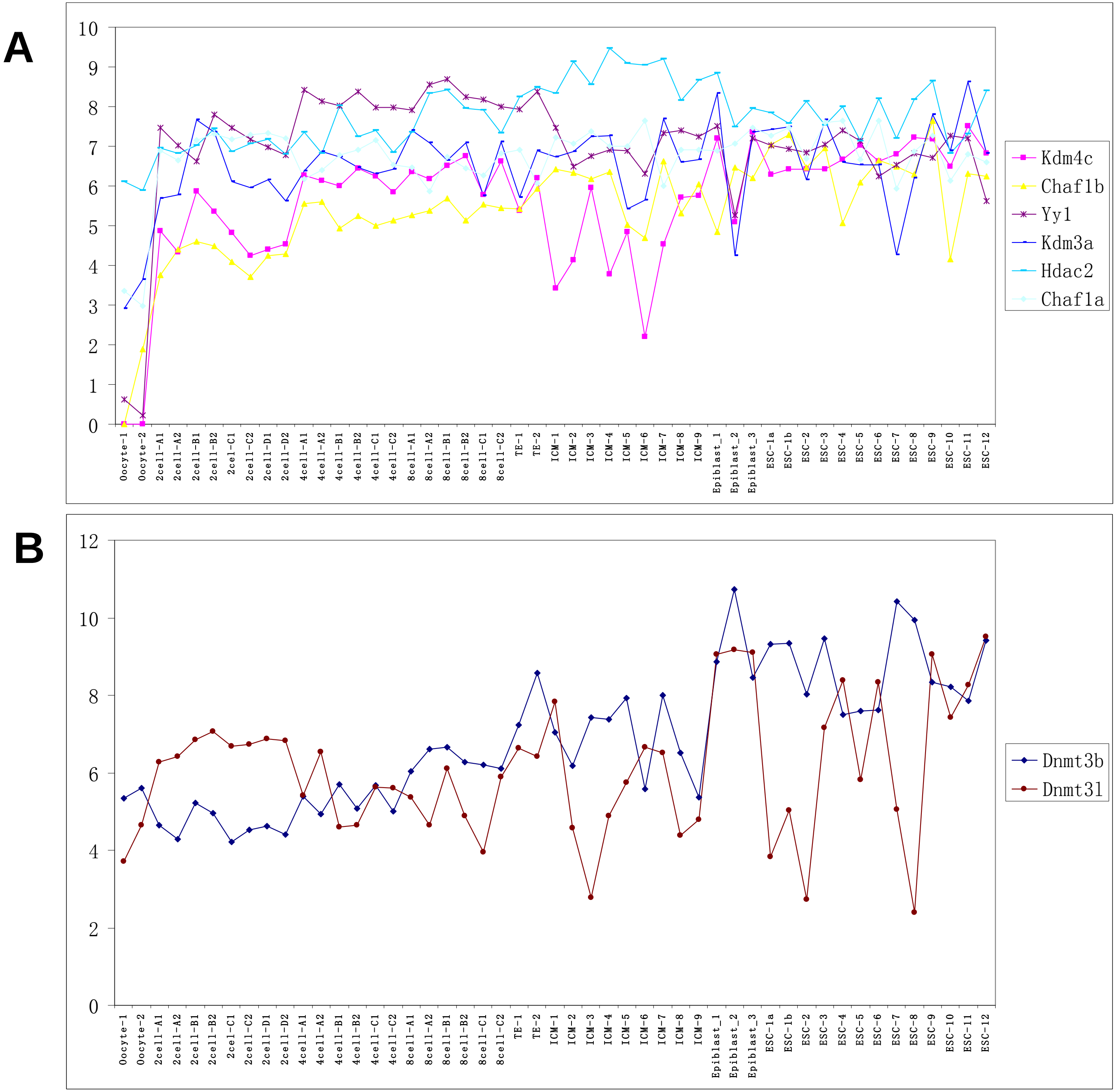

Figure S14

C

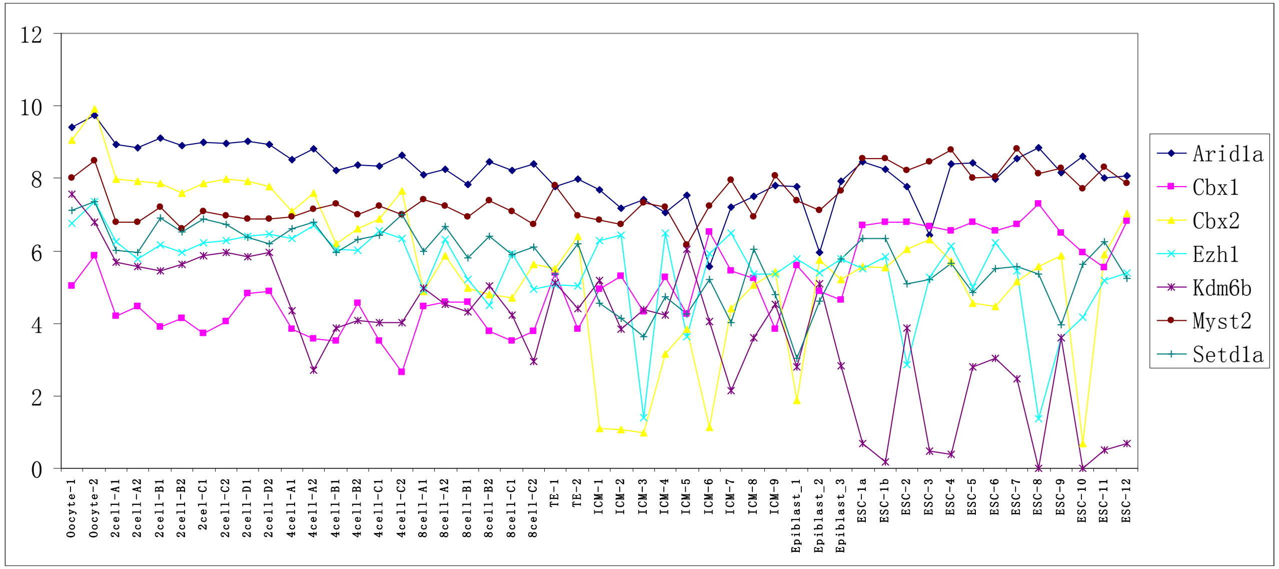

D

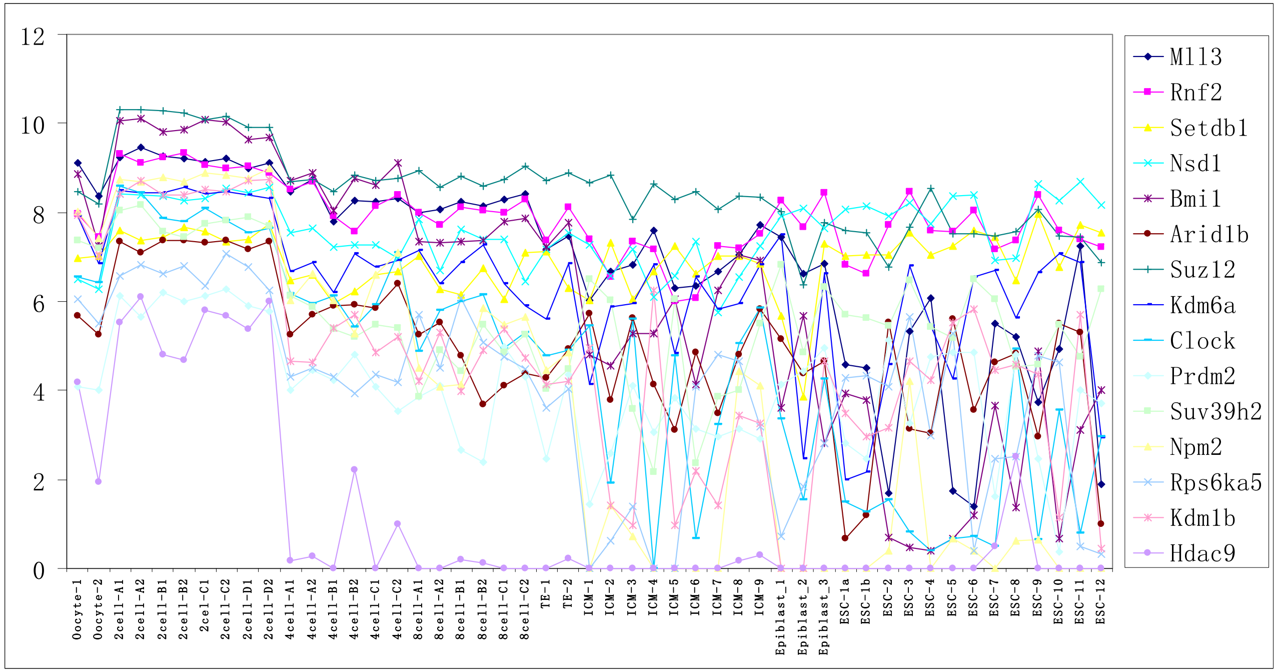

Figure S14

E

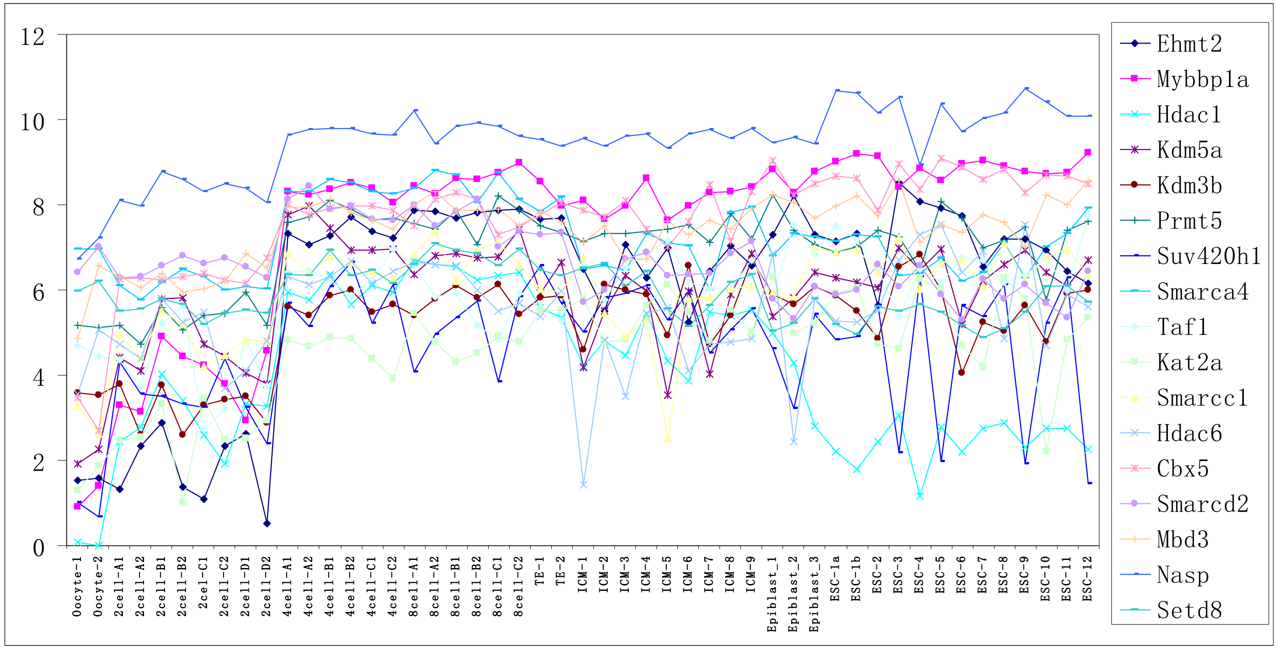

12
10
8
6
4
2
0
Oocyte-1
Oocyte-2
2cell-A1
2cell-A2
2cell-B1
2cell-B2
2cel-C1
2cell-C2
2cell-D1
2cell-D2
4cell-A1
4cell-A2
4cell-B1
4cell-B2
4cell-C1
4cell-C2
8cell-A1
8cell-A2
8cell-B1
8cell-B2
8cell-C1
8cell-C2
TE-1
TE-2
ICM-1
ICM-2
ICM-3
ICM-4
ICM-5
ICM-6
ICM-7
ICM-8
ICM-9
Epiblast_1
Epiblast_2
Epiblast_3
ESC-1a
ESC-1b
ESC-2
ESC-3
ESC-4
ESC-5
ESC-6
ESC-7
ESC-8
ESC-9
ESC-10
ESC-11
ESC-12
Ehmt2
Mybbp1a
Hdac1
Kdm5a
Kdm3b
Prmt5
Suv420h1
Smarca4
Taf1
Kat2a
Smarcc1
Hdac6
Cbx5
Smarcd2
Mbd3
Nasp
Setd8

Figure S14

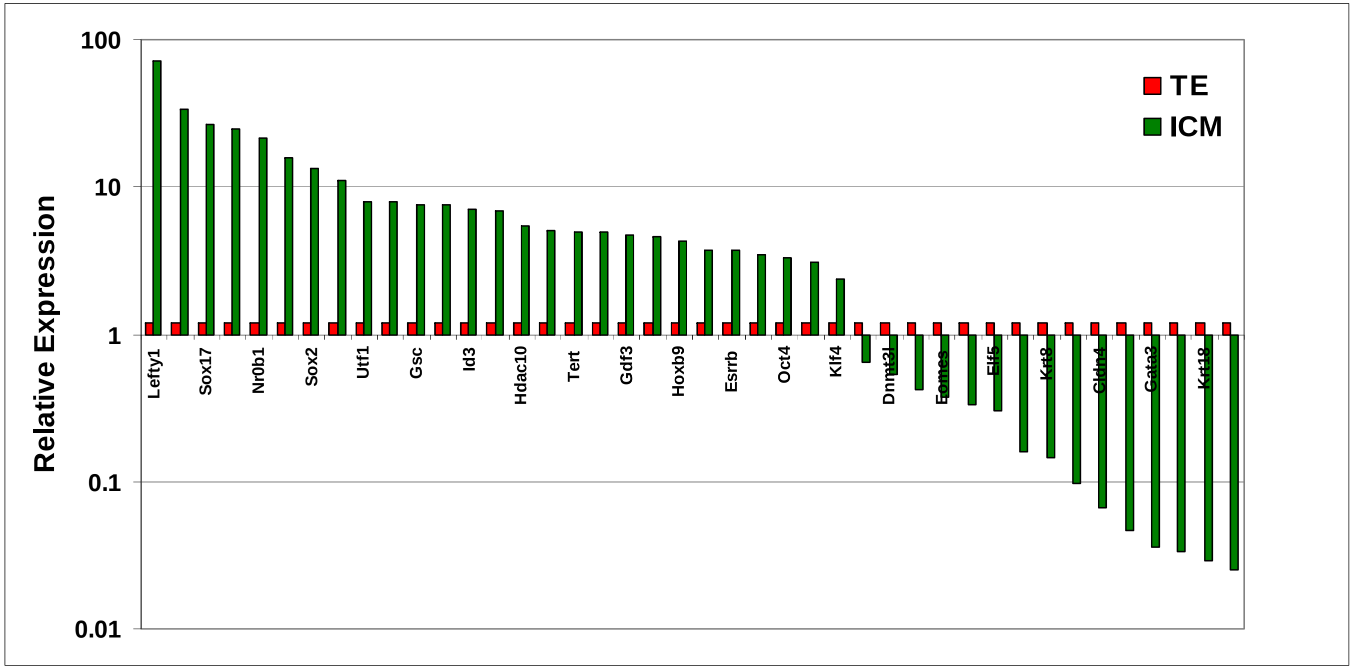
100
10
1
0.1
0.01
Relative Expression
TE
ICM
Lefty1
Sox17
Nr0b1
Sox2
Utf1
Gsc
Id3
Hdac10
Tert
Gdf3
Hoxb9
Esrrb
Oct4
Klf4
Dnmt3l
Eomes
Elf5
Krt8
Cldn4
Gata3
Krt18

Figure S15

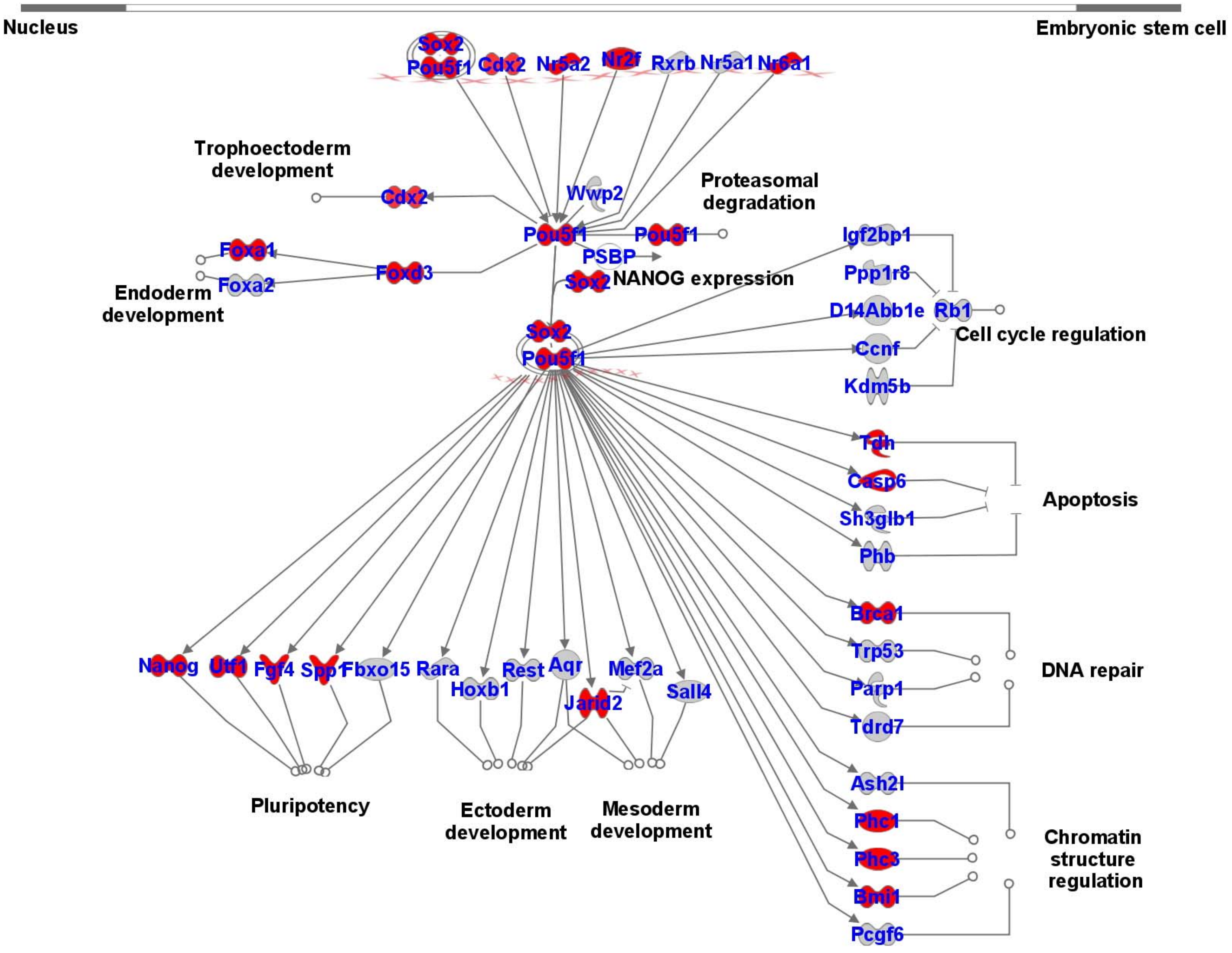
Nucleus
Embryonic stem cell
Sox2
Pou5f1
Cdx2
Nr5a2
Nr2f
Rxrb
Nr5a1
Nr6a1
Trophoectoderm development
Cdx2
Wwp2
Proteasomal degradation
Pou5f1
Pou5f1
Foxa1
Foxa2
Foxd3
Endoderm development
PSBP
Sox2
NANOG expression
Igf2bp1
Ppp1r8
D14Abb1e
Rb1
Ccnf
Kdm5b
Cell cycle regulation
Sox2
Pou5f1
Tdh
Casp6
Sh3glb1
Phb
Apoptosis
Brca1
Trp53
Parp1
Tdrd7
DNA repair
Nanog
Utf1
Fgf4
Spp1
Fbxo15
Rara
Hoxb1
Rest
Aqr
Jarid2
Mef2a
Sall4
Pluripotency
Ectoderm development
Mesoderm development
Ash2l
Phc1
Phc3
Bmi1
Pcgf6
Chromatin structure regulation

Figure S16

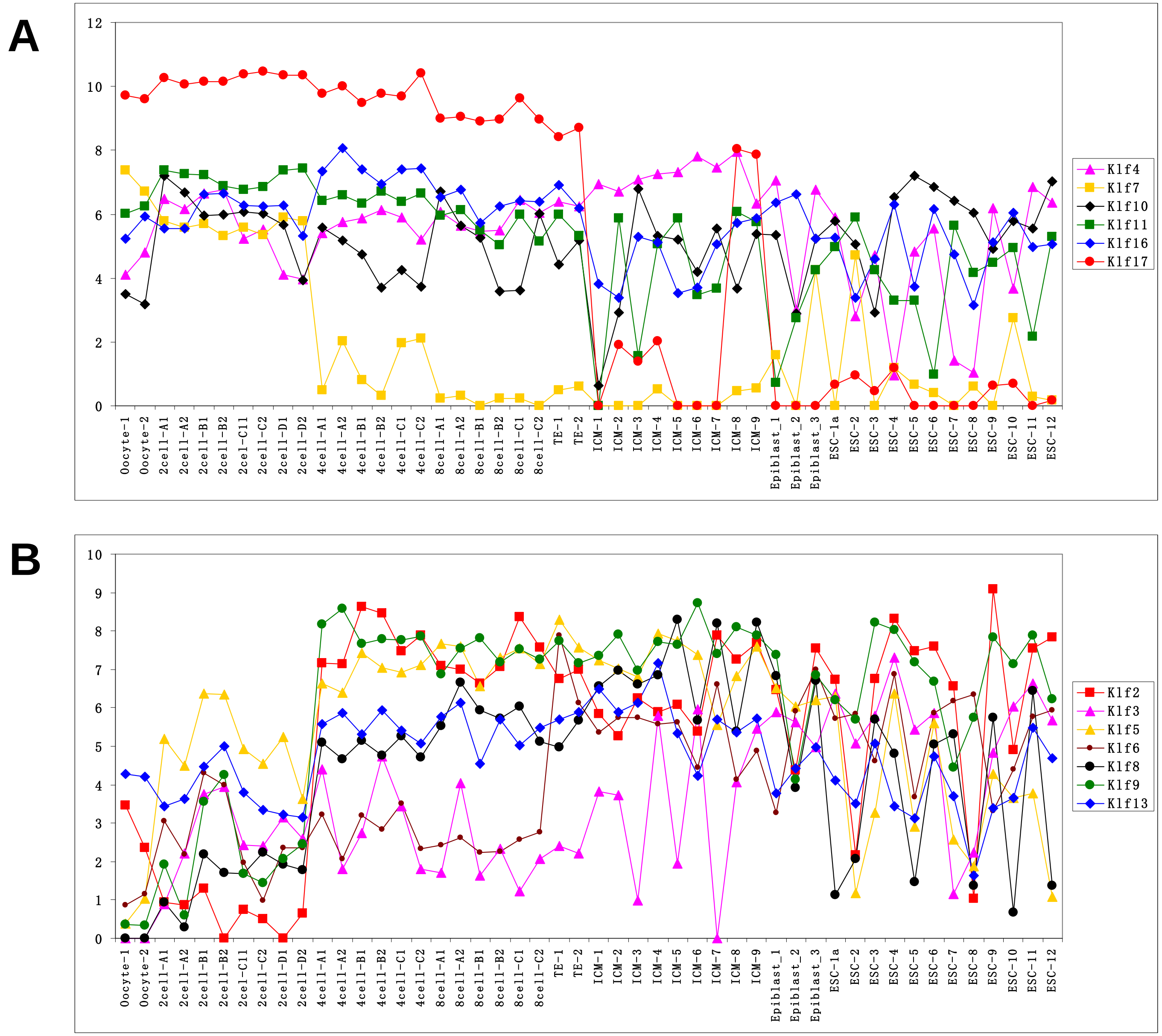
A
Klf4
Klf7
Klf10
Klf11
Klf16
Klf17
B
Klf2
Klf3
Klf5
Klf6
Klf8
Klf9
Klf13
Oocyte-1
Oocyte-2
2cell-A1
2cell-A2
2cell-B1
2cell-B2
2cel-C11
2cell-C2
2cell-D1
2cell-D2
4cell-A1
4cell-A2
4cell-B1
4cell-B2
4cell-C1
4cell-C2
8cell-A1
8cell-A2
8cell-B1
8cell-B2
8cell-C1
8cell-C2
TE-1
TE-2
ICM-1
ICM-2
ICM-3
ICM-4
ICM-5
ICM-6
ICM-7
ICM-8
ICM-9
Epiblast_1
Epiblast_2
Epiblast_3
ESC-1a
ESC-2
ESC-3
ESC-4
ESC-5
ESC-6
ESC-7
ESC-8
ESC-9
ESC-10
ESC-11
ESC-12

Figure S17

C

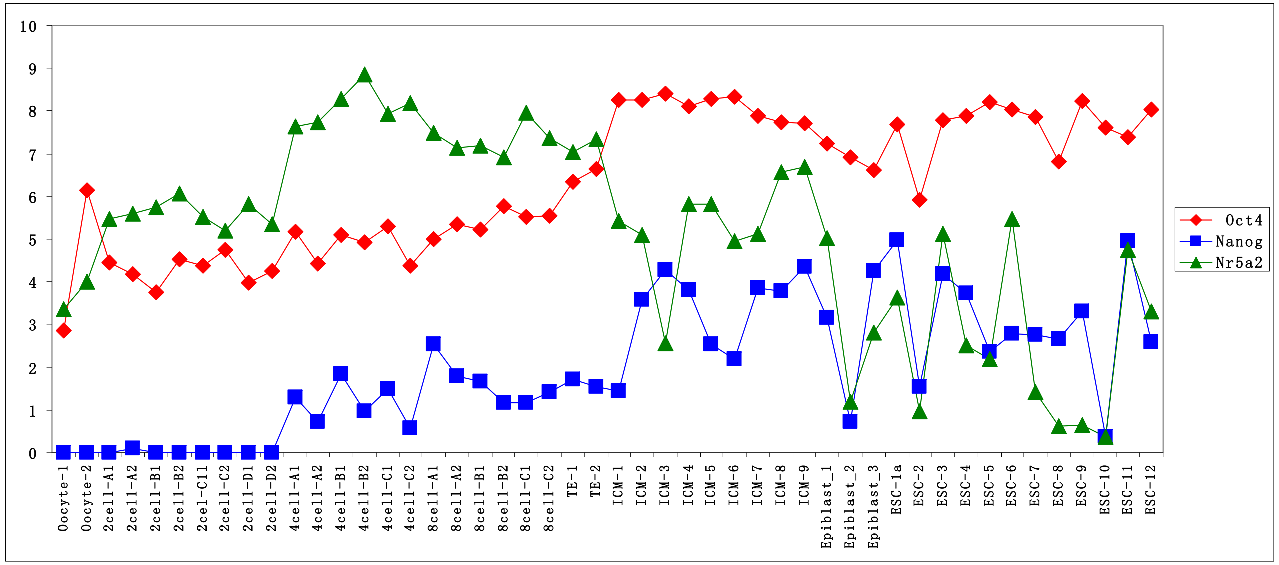

D

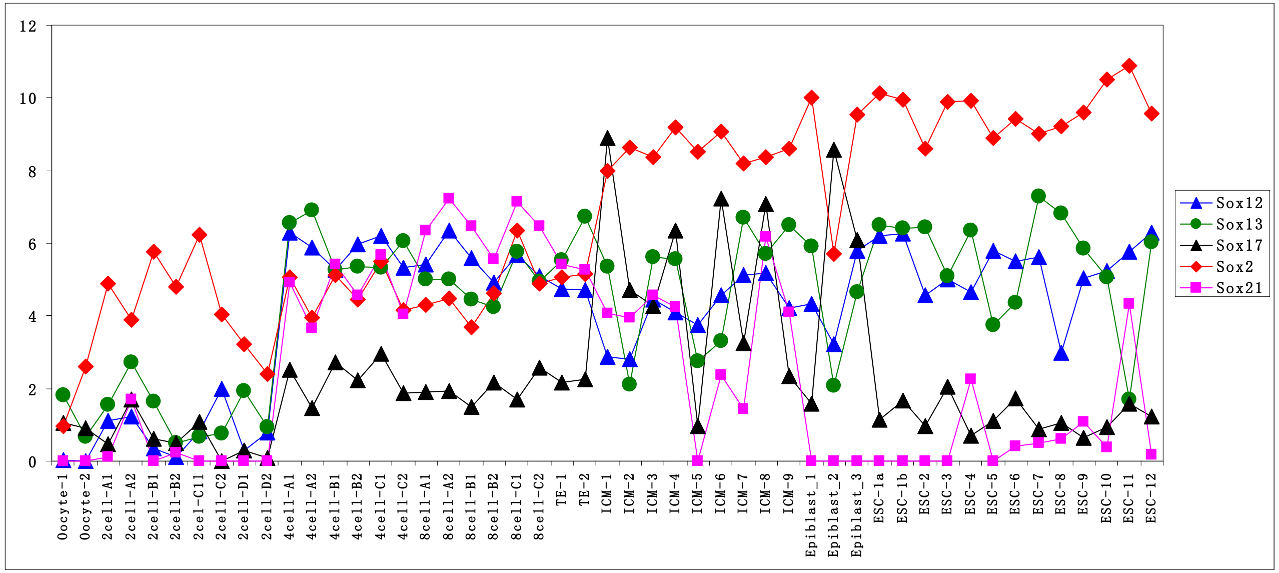

Figure S17

E

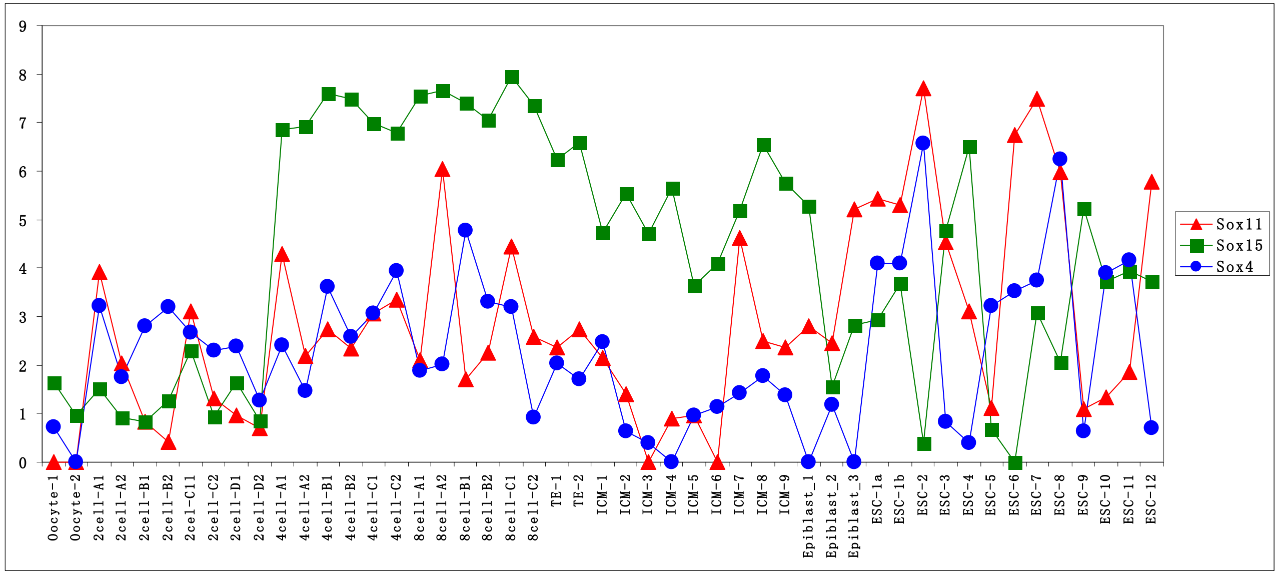

F

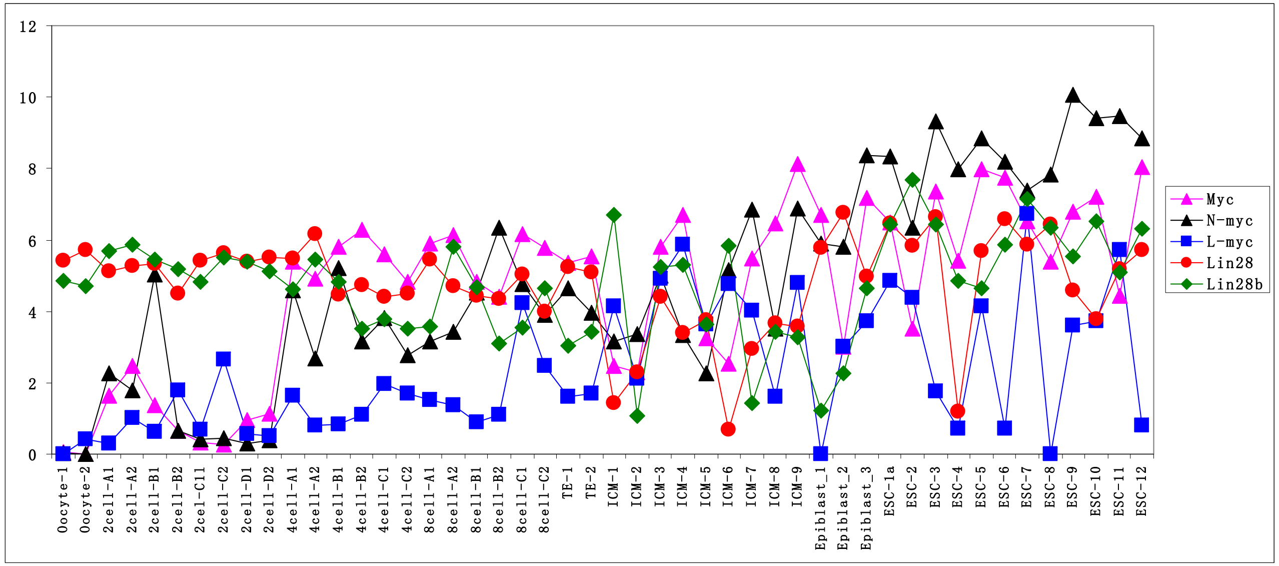

Figure S17
